# The short isoform of PRLR suppresses the pentose phosphate pathway and nucleotide synthesis through the NEK9-Hippo axis in pancreatic cancer

**DOI:** 10.7150/thno.51712

**Published:** 2021-02-06

**Authors:** Huizhen Nie, Pei-Qi Huang, Shu-Heng Jiang, Qin Yang, Li-Peng Hu, Xiao-Mei Yang, Jun Li, Ya-Hui Wang, Qing Li, Yi-Fan Zhang, Lei Zhu, Yan-Li Zhang, Yanqiu Yu, Gary Guishan Xiao, Yong-Wei Sun, Jianguang Ji, Zhi-Gang Zhang

**Affiliations:** 1 State Key Laboratory of Oncogenes and Related Genes, Shanghai Cancer Institute, Ren Ji Hospital, School of Medicine, Shanghai Jiao Tong University, Shanghai, P.R. China.; 2 Department of Pathophysiology, College of Basic Medical Sciences, China Medical University, Shenyang, P.R. China.; 3 School of Pharmaceutical Science and Technology, Dalian University of Technology, Dalian, P.R. China.; 4 Functional Genomics and Proteomics Laboratory, Osteoporosis Research Center, Creighton University Medical Center, Omaha, Nebraska.; 5 Department of Biliary-Pancreatic Surgery, Ren Ji Hospital, School of Medicine, Shanghai Jiao Tong University, Shanghai, P.R. China.; 6 Center for Primary Health Care Research, Department of Clinical Sciences, Malmö Lund University, Lund, Sweden.

**Keywords:** isoform, pancreas, hormone, metabolism, biosynthesis.

## Abstract

Prolactin binding to the prolactin receptor exerts pleiotropic biological effects in vertebrates. The prolactin receptor (PRLR) has multiple isoforms due to alternative splicing. The biological roles and related signaling of the long isoform (PRLR-LF) have been fully elucidated. However, little is known about the short isoform (PRLR-SF), particularly in cancer development and metabolic reprogramming, a core hallmark of cancer. Here, we reveal the role and underlying mechanism of PRLR-SF in pancreatic ductal adenocarcinoma (PDAC).

**Methods:** A human PDAC tissue array was used to investigate the clinical relevance of PRLR in PDAC. The *in vivo* implications of PRLR-SF in PDAC were examined in a subcutaneous xenograft model and an orthotopic xenograft model. Immunohistochemistry was performed on tumor tissue obtained from genetically engineered KPC (Kras^G12D/+^; Trp53^R172H/+^; Pdx1-Cre) mice with spontaneous tumors. ^13^C-labeled metabolite measures, LC-MS, EdU incorporation assays and seahorse analyses were used to identify the effects of PRLR-SF on the pentose phosphate pathway and glycolysis. We identified the molecular mechanisms by immunofluorescence, coimmunoprecipitation, proximity ligation assays, chromatin immunoprecipitation and promoter luciferase activity. Public databases (TCGA, GEO and GTEx) were used to analyze the expression and survival correlations of the related genes.

**Results:** We demonstrated that PRLR-SF is predominantly expressed in spontaneously forming pancreatic tumors of genetically engineered KPC mice and human PDAC cell lines. PRLR-SF inhibits the proliferation of PDAC cells (AsPC-1 and BxPC-3) *in vitro* and tumor growth *in vivo*. We showed that PRLR-SF reduces the expression of genes in the pentose phosphate pathway (PPP) and nucleotide biosynthesis by activating Hippo signaling. TEAD1, a downstream transcription factor of Hippo signaling, directly regulates the expression of G6PD and TKT, which are PPP rate-limiting enzymes. Moreover, NEK9 directly interacts with PRLR-SF and is the intermediator between PRLR and the Hippo pathway. The PRLR expression level is negatively correlated with overall survival and TNM stage in PDAC patients. Additionally, pregnancy and lactation increase the ratio of PRLR-SF:PRLR-LF in the pancreas of wild-type mice and subcutaneous PDAC xenograft tumors.

**Conclusion:** Our characterization of the relationship between PRLR-SF signaling, the NEK9-Hippo pathway, PPP and nucleotide synthesis explains a mechanism for the correlation between PRLR-SF and metabolic reprogramming in PDAC progression. Strategies to alter this pathway might be developed for the treatment or prevention of pancreatic cancer.

## Introduction

PRLR is a member of the cytokine receptor superfamily. PRLR is expressed in almost all tissues and organs and has many functions 1-3. In addition to its crucial role in lactation, many other functions have recently been attributed to PRLR, including roles in metabolism, bone homeostasis, maternal care, stress and adrenal function, electrolyte transport, immunity and carcinogenesis 1, 2. In mammals, *PRLR* contains at least 10 exons, and alternative splicing produces different isoforms. These isoforms have an identical extracellular domain but differ in the size and sequence of the intracellular portion—they can be short, intermediate, or long. All of these isoforms may regulate different signaling pathways 4.

The human long PRLR (PRLR-LF), considered the major isoform through which prolactin transmits its signals, has an apparent mass of 90 kDa and is composed of 598 amino acids. PRLR-LF is the primary receptor in breast and most other tissues, where it functions through STAT5, PI3K/AKT and the Ras-Raf-Mek-MAPK pathway 2, 5.

Alternative splicing and deletion generate multiple short human PRLR isoforms (PRLR-SFs). Both the SF1a and SF1b isoforms are spliced into exon 11. The SF1a isoform has 376 amino acids and includes part of exon 10 and 39 amino acids from exon 11, whereas S1b lacks exon 10 and contains only three residues from exon 11 6, 7. Dufau *et al.* showed that human PRLR-SF (SF1a and SF1b) could also activate ligand-dependent Jak2 phosphorylation 8. In contrast, Clevenger 9 reported those rats PRLR-SF homodimers are unable to activate Jak2. Their work emphasizes the importance of tyrosine phosphorylation at the Y309 and Y382 residues for the activation of Jak2, regions that are absent in the PRLR-SF. Another group has shown that the box2 region, which is present in PRLR-LF but not in PRLR-SF, is required for Jak2 activation 10, 11.

In prostate cancer cells, upregulated expression of PRLR-SF1b increases both the expression of growth inhibitor p21 and the vitamin D receptor 12. In female rat reproductive tissues, the proportion of PRLR-LF: PRLR-SF receptors vary depending on hormonal fluctuations, whereas the liver maintains a constant preference for rat PRLR-SF 13, 14. PRLR-SF1a has been demonstrated to constrain tumor-promoting liver inflammation by inhibiting MAP3K-dependent activation of c-Myc at the level of the TRAFasome 15. PRLR-SF is generally regarded as a negative regulator 7, 16, 17.

The cytoplasmic region of mouse PRLR is encoded by exon 10 for the long PRLR, exon 12 for PR-1, exon 11 for PR-2, and exon 13 for PR-3, but only two short forms have been identified as encoding proteins 6, 7. In transgenic mice expressing only PRLR-SF (PR-3), the MAPK pathway is deactivated in the ovary and decidua 16. PRLR-SF (PR-3) does not trigger JAK signaling to activate STAT but instead reduces the expression of SP1, FOXO3, and GALT 10, 18.

Expression of both PRLR-SF1a and PRLR-SF1b mRNAs is obviously detectable in samples of human liver, pancreas, placenta and kidney 19. The liver and pancreas are solid tissues that arise from the endoderm during vertebrate development. In addition to similarities in function and morphology in adulthood, the liver and pancreas share a common developmental history, a set of early morphological patterning events and similar early transcription factors 20. In rats, liver PRLR includes up to 92% mRNA encoding short PRLR. Although not as high as in the liver, the percentage of short PRLR (38%) in the pancreas is among the mid-range expression tissues (kidney, adrenal, spleen, thymus) 19, 21. Pancreatic ductal adenocarcinoma (PDAC) is the fourth leading cause of cancer-related deaths in developed countries, with a 5-year rate of survival below 8% 22, 23. Researchers have studied PRLR signaling in PDAC cells 24, 25, but the mechanism by which PRLR regulates cell proliferation is not well understood. Moreover, little is known about prolactin signaling via PRLR-SF or the contribution of this pathway to pancreatic tumor progression.

Here, we identified, for the first time, that PRLR-SF reduces nucleotide synthesis in PDAC cells via the Hippo pathway, which inhibits the pentose phosphate pathway (PPP) to prevent PDAC cell proliferation and tumor growth. Moreover, NEK9 (NIMA-related kinase 9) is the intermediator between PRLR and Hippo signaling. PRLR-SF exerts a tumor-suppressive role in PDAC by preventing PPP metabolism.

## Materials and methods

### Plasmids and antibodies

The human PRLR S1a isoform ORF (NM_001204316.1) was synthesized and cloned into the pReceiver-Lv241 vector (with Flag tag). The sh-RNA of human PRLR was cloned into plenti-shRNA-GFP-Puro. The human NEK9 ORF (NM_001329237.2) was cloned into the pReceiver-M07 vector (with HA tag). The human G6PD ORF (NM_000402.4) and TKT ORF (NM_001258028.2) were cloned into the pReceiver-M02 vector. The human YAP ORF (NM_001130145.2) was subcloned into the pcDNA3.1 vector to generate the pcDNA3.1-YAP S127A and -YAP△PDZ plasmids, respectively. The YAP S127A mutant cannot be phosphorylated by LATS kinases and is thus located in the cell nucleus. The YAP△PDZ mutant lacks the nuclear shuttling PDZ domain, the five most C-terminal amino acids FLTWL, and thus is sequestered to the cytoplasm 26.

The immunogen of PRLR antibody (35-9200, Thermo) is recombinant human prolactin receptor protein containing the extracellular domain (ECD). The PRLR antibody (MAB1167-SP, NOVUS) was used in the neutralizing experiment, and the immunogen was the mouse myeloma cell line NS0-derived recombinant human PRLR Gln25-Asp234 27. Additional antibody information is available in the Supplementary Materials and Methods.

### Clinical samples

Tissue microarrays containing 311 PDAC specimens and PDAC tissues were obtained from Ren Ji Hospital from January 2002 to June 2015. All patients provided written informed consent before enrollment, and the study was approved by the Research Ethics Committee of Ren Ji Hospital, School of Medicine, Shanghai Jiao Tong University.

### Transgenic animal model

The Pdx1-Cre and LSL-Trp53^R172H/+^ mice were all obtained from The Jackson Laboratory (https://www.jax.org) (LSL: Lox/Stop/Lox). The LSL-Kras^G12D/+^ mice were a generous gift from Professor Xiu-Feng Pang (Shanghai Key Laboratory of Regulatory Biology, Institute of Biomedical Sciences and School of Life Sciences, East China Normal University). The Pdx1-Cre, LSL-Kras^G12D/+^, and LSL-Trp53^R172H/+^ mice were raised in our laboratory. The KP mice were generated by hybridizing the LSL-Kras^G12D/+^ mice and LSL-Trp53^R172H/+^ mice. The KPC mice were generated by hybridizing the LSL-Kras^G12D/+^ mice, LSL-Trp53^R172H/+^ mice and Pdx1-Cre mice. All animal experiments were undertaken in accordance with the NIH Guide for the Care and Use of Laboratory Animals.

### mRNA microarray

Lenti-vector/AsPC-1 and Lenti-PRLR-SF/AsPC-1 cells were collected and homogenized in TRIzol (Invitrogen). A complementary DNA (cDNA) microarray analysis was performed by Shanghai Biotechnology Corporation. The transcript profiling of the Lenti-vector/AsPC-1 and Lenti-PRLR-SF/AsPC-1 cells was submitted to the National Center for Biotechnology Information's GEO database, and the repository URL and the data accession numbers are GSE159917.

### LC-MS analysis of the cell metabolites

Cells with various treatments were cultured with Dulbecco's modified Eagle's medium (DMEM, Gibco, A14430-01) containing 4.5 g/L glucose, half of which was U-^13^C labeled (CIL, CLM-1396-1), for 1 hr. Then, 5 × 10^6^ cells were washed twice with cold PBS. After centrifugation, ice-cold extraction buffer (methanol: acetonitrile: H_2_O = 2:2:1) was immediately added to the cell pellets at five volumes. Samples were treated with freeze and thaw cycles (freeze in liquid nitrogen for 1 min and thaw at room temperature) at least three times to lyse the cells sufficiently. Then, the same volume of chloroform was added to the samples and vortexed for 10 s. The mixtures were centrifuged at 12000 rpm for 15 min, and the supernatant was collected and dried. The powder containing the metabolites was dissolved in 80% methanol before running the LC-MS.

### Seahorse Analyses

The assays detecting the extracellular acidification rate (ECAR) and oxygen consumption rate (OCR) of cultured cells were performed with the Seahorse XF96 Flux Analyzer (Seahorse Bioscience, Agilent) as previously described 28. Briefly, AsPC-1 or BxPC-3 cells were seeded in an XF96-well plate at 4×10^4^ cells per well or 2×10^4^ per well, respectively, with the indicated treatment. The culture media was replaced with assay media 1 hr before detection. For the glycolytic stress test (Seahorse Cat. #103020-100), 10 mM glucose, 1 μM oligomycin and 50 mM 2-deoxyglucose (2-DG) were injected into the wells in that order. For the mitochondrial stress test (Seahorse Cat. #103015-100), 1 μM oligomycin, 1 μM FCCP, 0.5 μM rotenone and 0.5 μM actinomycin A were injected into the wells in that order. Both measurements were normalized by total protein quantitation. The above experiments were performed in triplicate and repeated twice.

### ChIP-PCR assay

For the ChIP-PCR assay, according to the procedures previously described 29, the cells were cross-linked and sonicated, and the DNA was immunoprecipitated with anti-TEAD1 (GTX32918; Gene Tex, Irvine, CA) or isotype-matched control IgG (CST) from the lysates and quantified using Premix Taq PCR analysis (TaKaRa).

### Immunoprecipitation

Cells were transfected with HA-tagged NEK9, Flag-tagged PRLR or vector control and lysed for immunoprecipitation. The lysates were incubated with anti-HA or anti-Flag antibody or control IgG coupled beads and rotated overnight at 4 ℃. After washing the beads with the prepared lysis buffer, 1× SDS loading buffer was added to the beads, boiled for 5 min, and then analyzed by western blotting.

### *In situ* proximity ligation assay

An *in situ* proximity ligation assay (PLA, Sigma, DUO92007) was performed as previously described 30. Briefly, AsPC-1 and BxPC-3 cells were seeded in a 12-well chamber overnight, fixed for 30 mins at room temperature, and then permeabilized with 0.1% Triton-X100 for 2 mins. After incubation with DuoLink blocking buffer for 60 min at 37 ℃, the cells were incubated with primary antibodies against PRLR and NEK9 from two different species at 4 ℃ overnight. Next, the cells were incubated with DuoLink PLA MINUS and PLUS Probes at 37 ℃ for 1 hr, and then ligation solution was added to form a closed circle at 37 ℃ for 30 mins. Next, the amplification reaction was performed by polymerase at 37 ℃ for 100 mins. Finally, the cells were stained with DAPI and mounted. All the assays were performed under humidified conditions to avoid false-positive results.

Additional protocols and procedures are described in the supplementary materials and methods.

### Statistical analysis

Statistical analysis and graphical representations were performed using GraphPad Prism 7.0. Data are presented as the means ± standard deviation. Student's t test and two-way ANOVA were used for comparisons between groups. Cumulative survival curves were evaluated using the Kaplan-Meier method, and differences between the survival curves were tested by the log-rank test. Values of P < 0.05 were considered statistically significant.

## Results

### PRLR-SF contributes to proliferation inhibition in PDAC cells

**Figure 1:**
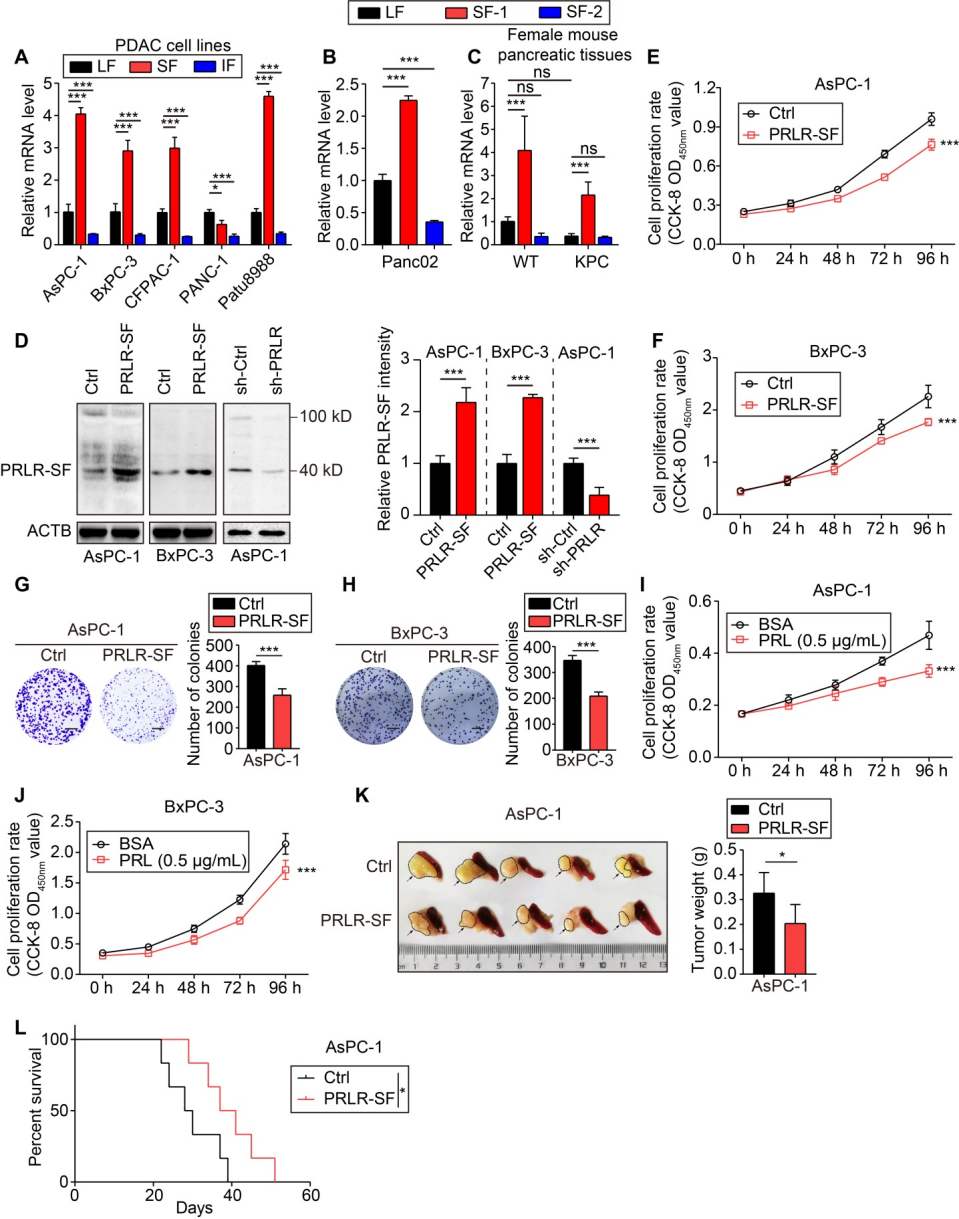
** PRLR-SF contributes to proliferation inhibition in PDAC cells.** (**A**) Real-time qPCR analyses for expression level of long isoform (LF), short isoform (SF) and intermediate isoform (IF) of PRLR in human PDAC cell lines. (**B**, **C**) Real-time qPCR analyses for expression level of long isoform (LF), short isoform 1 (SF-1, PR3) and short isoform 2 (SF-2, PR1) of Prlr in mouse PDAC cell line Panc02 and pancreatic tissues from wild type (WT) and LSL-Kras^G12D/+^; LSL-Trp53^R127H/+^; Pdx1-cre (KPC) female mice, respectively. (**D**) Left, immunoblots of PRLR protein in AsPC-1 cells and BxPC-3 cells stably expressing vector only (Ctrl) or a vector that encodes for PRLR-SF, and in AsPC-1 cells infected with sh-Ctrl or sh-PRLR. Right, quantification of PRLR-SF intensity, n = 3 each group, *P* values were determined by unpaired *t* test. (**E**,** F**) Proliferation of AsPC-1 cells (**E**) and BxPC-3 cells (**F**) stably expressing Ctrl vs overexpression of PRLR-SF (n = 5). *P* values were determined by 2-way ANOVA. (**G, H**) Clonogenicity of Ctrl AsPC-1 vs AsPC-1 that overexpress PRLR-SF (**G**) and Ctrl BxPC-3 vs BxPC-3 that overexpress PRLR-SF (**H**) (n = 3); scale bar=500 mm. *P* values were determined by unpaired *t* test. (**I, J**) Proliferation of AsPC-1 cells (**I**) and BxPC-3 cells (**J**) incubated with 0.1%BSA or prolactin (PRL, 0.5μg/ml in 0.1% BSA) for 96 hrs (n = 5).* P* values were determined by 2-way ANOVA. (**K**) Tumors with adjacent pancreas and spleen from orthotopic tumors grown in nude mice from implanted Ctrl AsPC-1 or AsPC-1 cells that overexpress PRLR-SF; tumor weight is shown (n = 5). *P* values were determined by unpaired *t* test. (**L**) Kaplan-Meier survival analysis of nude mice with orthotopic tumors grown from implanted control (Ctrl) AsPC-1 cells or cells that overexpress PRLR-SF. *P* value was calculated with log-rank (Mantel-Cox) test. *P < 0.05, ***P < 0.001.

To investigate the expression of the PRLR isoforms in PDAC cells, we designed primers to measure the expression of PRLR-SF and PRLR-LF in spontaneously forming pancreatic tumors from genetically engineered KPC mice and human PDAC cell lines. We found that, compared to PRLR-LF, PRLR-SF is predominantly expressed in pancreatic tumors of the KPC mice, the mouse PDAC cell line (Panc02) and the human PDAC cell lines (AsPC-1, BxPC3, CFPAC-1, and Patu8988) (Figure 1A-C).

Next, we performed functional studies of 2 cell lines with relatively low levels of *PRLR-LF* mRNA but high levels of *PRLR-SF* mRNA (AsPC-1 and BxPC-3 cells). Immunoblot analyses of PDAC tissues revealed that the molecular weight of the most highly expressed form of PRLR-SF was 40 KD (Figure S1A); therefore, we overexpressed SF1a (40 KD), not SF1b (32 KD), in PDAC cells. Lentivirus-mediated stable PRLR-SF overexpression and knockdown of PRLR-SFs were validated by immunoblotting and quantitative PCR (Figure 1D, Figure S1B). We found that prolactin was expressed in the PDAC cells (Figure S1C). Cell proliferation and colony formation were reduced by the overexpression of PRLR-SF and were promoted by treatment with siRNA and shRNA against PRLR in the PDAC cell lines (Figure 1E-H, Figure S1D-J). The proliferation of AsPC-1 and BxPC-3 cells incubated with prolactin (PRL) was also reduced (Figure 1I-J). The addition of a blocking antibody against PRLR restored cell proliferation to previous levels (Figure S2E). These data indicate that the activation of PRLR-SF by its physiologic ligand (prolactin) contributes to the proliferation inhibition of PDAC cells.

We further investigated the tumor-suppressive role of PRLR-SF *in vivo* using subcutaneous and orthotopic transplantation. PDAC cell lines that overexpressed PRLR-SF formed significantly smaller xenograft tumors in mice (based on the tumor weights, see Figure 1K and Figure S3B) than PDAC cells transduced with a control vector and had a lower proliferation index (based on staining for proliferating cell nuclear antigen, see Figure S3A). The PRLR-SF overexpression group showed extended overall survival compared with the control group in the orthotopic mouse model (Figure 1L). The cells treated with shPRLR formed significantly larger xenograft tumors in the mice than the control cells (Figure S3C). In the mice with subcutaneous xenograft tumors, continuous injection of prolactin into the tumors for 10 days significantly decreased the tumor weights (Figure S3D). These data indicate that PRLR-SF overexpression reduced tumor growth in mice. Altogether, these results reveal that PRLR-SF overexpression reduces the proliferation and colony formation of PDAC cells and reduces their growth as xenograft tumors in mice, suggesting a tumor-suppressive role of PRLR-SF. Using an *in vitro* assay, Utama *et al.* showed that mouse prolactin and growth hormone were less potent than human prolactin binding to human PRLR 31. However, in our *in vivo* long-term animal experimental setting of orthotopic xenograft, the effects of PRL lasted for 4 weeks continuously. This might provide an explanation for the protective role of prolactin against PDAC in animal models.

### PRLR-SF downregulates genes in the phosphate pentose pathway and biosynthesis

**Figure 2:**
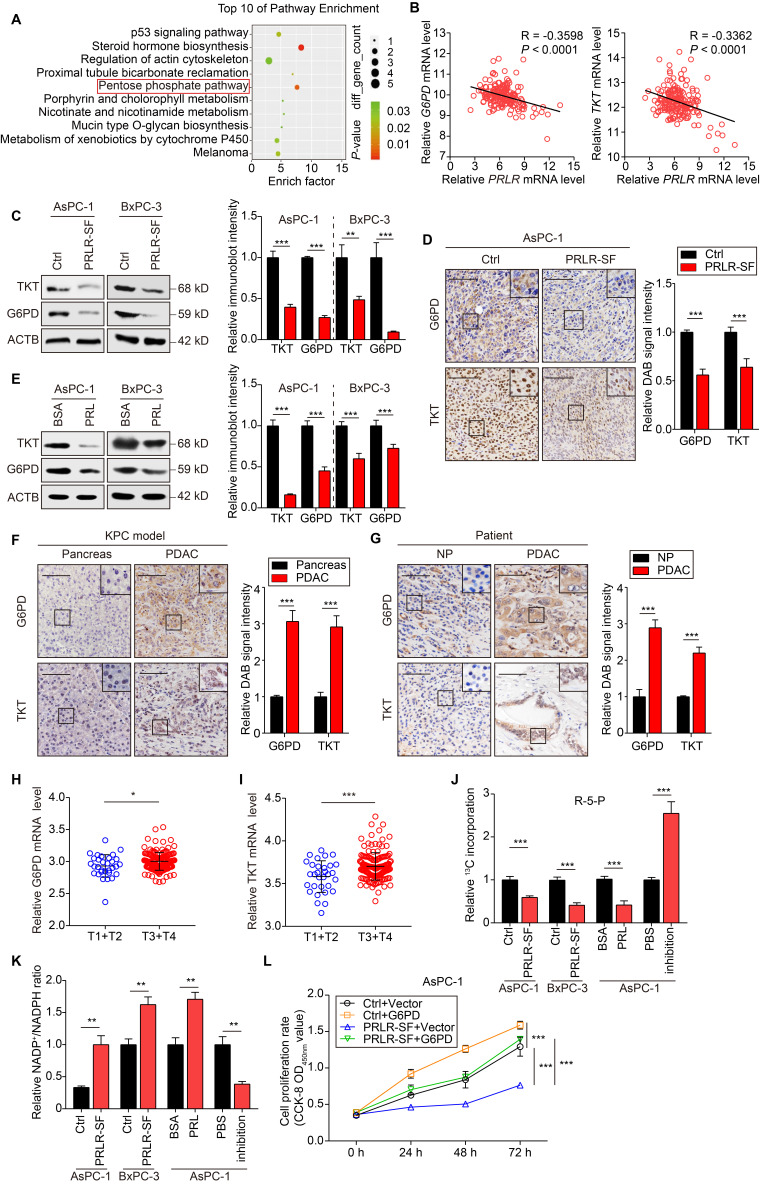
** PRLR-SF inhibits PPP biosynthesis by reducing G6PD and TKT expression in PDAC cells.** (**A**) GO analyses of whole-genome expression profiles of AsPC-1-Ctrl cells vs AsPC-1-PRLR-SF demonstrates increased expression of genes in the PPP. (**B**) Linear regression analyses for *PRLR* vs *G6PD* (left) or *TKT* (right) mRNAs in PDAC tumor tissues, using TCGA data (n = 182). Pearson correlation coefficients (R) are shown. (**C**) Left, immunoblot analyses of G6PD and TKT proteins in control (Ctrl) AsPC-1 cells and BxPC-3 cells and cells that overexpress PRLR-SF. Right, immunoblotting quantification of G6PD and TKT, n = 3 each group, *P* values were determined by unpaired *t* test. (**D**) Left, representative images of immunohistochemical analyses of levels of G6PD (top) and TKT (bottom) in xenograft tumors grown from AsPC-1-Ctrl cells or AsPC-1-PRLR-SF cells. Right, DAB signal quantification of G6PD and TKT, n = 3 per group, *P* values were determined by unpaired *t* test. (**E**) Left, immunoblot analyses of G6PD and TKT proteins in AsPC-1 cells and BxPC-3 cells incubated with 0.1%BSA or prolactin (PRL, 0.5μg/ml in 0.1% BSA). Right, immunoblotting quantification of G6PD and TKT, n = 3 per group, *P* values were determined by unpaired *t* test. (**F**, **G**) Left, representative images of immunohistochemical analyses of levels of G6PD (top) and TKT (bottom) pancreatic tissues from KPC mice (**F**), and pancreatic tissues from a patient (**G**). NP, paired non-PDAC, Scale bar =100 μm. Right, DAB signal quantification of G6PD and TKT, n = 3 per group, *P* values were determined by unpaired *t* test. (**H**, **I**) Relative *G6PD* (**H**) and *TKT* (**I**) mRNA level for T1+T2 stages and T3+T4 stages of patients based on TCGA data. *P* values were determined by unpaired *t* test. (**J**,** K**) Incorporation of ^13^C-labeled ribose-5-phostate (R-5-P) and (**J**) and ratios of NADP^+^ to NADPH (**K**) in AsPC-1 cells and BxPC-3 cells overexpressing PRLR-SF vs control (Ctrl) cells, and AsPC-1 cells incubated with 0.1%BSA, prolactin (PRL), phosphate-buffered saline (PBS), or antibody against PRLR (inhibition). (**L**) Proliferation of AsPC-1 cells infected with Lenti-control (Ctrl) or Lenti-PRLR-SF, and transfected transiently with empty vectors (vector) or vectors that overexpress G6PD (n = 5). *P* values determined by 2-way ANOVA. *P < 0.05, **P < 0.01, ***P < 0.001.

To explore how PRLR-SF inhibits tumor growth, we performed genome-wide cDNA microarray analyses of control AsPC-1 cells (AsPC-1-Ctrl cells) and cells that overexpress PRLR-SF (AsPC-1-PRLR-SF cells; see Figure S4A). Pathway enrichment analysis of the transcriptome data revealed alterations in the PPP (Figure 2A). The PPP plays a critical role in macromolecule biosynthesis and in maintaining cellular redox homeostasis in rapidly proliferating cells 32, 33. We performed further analyses of mRNAs encoding 7 proteins in the PPP (G6PD, PGD, PGLS, TALDO1, TKT, RPE, and RPIA) (Figure S4B). Of these, *G6PD* (glucose-6-phosphate dehydrogenase) and *TKT* (transketolase) mRNAs were significantly reduced in AsPC1-PRLR-SF cells compared to AsPC-1-Ctrl cells. Knockdown of G6PD and TKT significantly suppressed DNA synthesis in PDAC cells (Figure S4C-F). In an analysis of the Cancer Genome Atlas (TCGA) database, we found that levels of *PRLR* mRNA correlated inversely with the levels of *G6PD* and *TKT* mRNAs in PDAC tissues (Figure 2B). We confirmed reduced levels of G6PD and TKT proteins in PDAC cells that overexpressed PRLR-SF (Figure 2C) and xenograft tumors (Figure 2D). When the PDAC cells were incubated with prolactin, the levels of the G6PD and TKT proteins decreased (Figure 2E). These data indicate that PRLR-SF expression reduces the expression of two key enzymes in PPP, G6PD and TKT, in PDAC cells.

We used immunohistochemistry (IHC) to analyze the expression patterns of G6PD and TKT in pancreatic tissues from genetically engineered spontaneous tumor-producing KPC mice and human PDAC tissues. G6PD and TKT were more highly expressed in pancreatic tumors from KPC mice than in pancreatic tissues from WT mice (Figure 2F). The levels of G6PD and TKT were significantly increased in human PDAC tissues compared with the corresponding paracancerous tissues (Figure 2G). TCGA database analysis showed that the mRNA levels of G6PD and TKT were positively correlated with PDAC T stage, indicating that G6PD and TKT might promote PDAC progression (Figure 2H, I).

**Figure 3:**
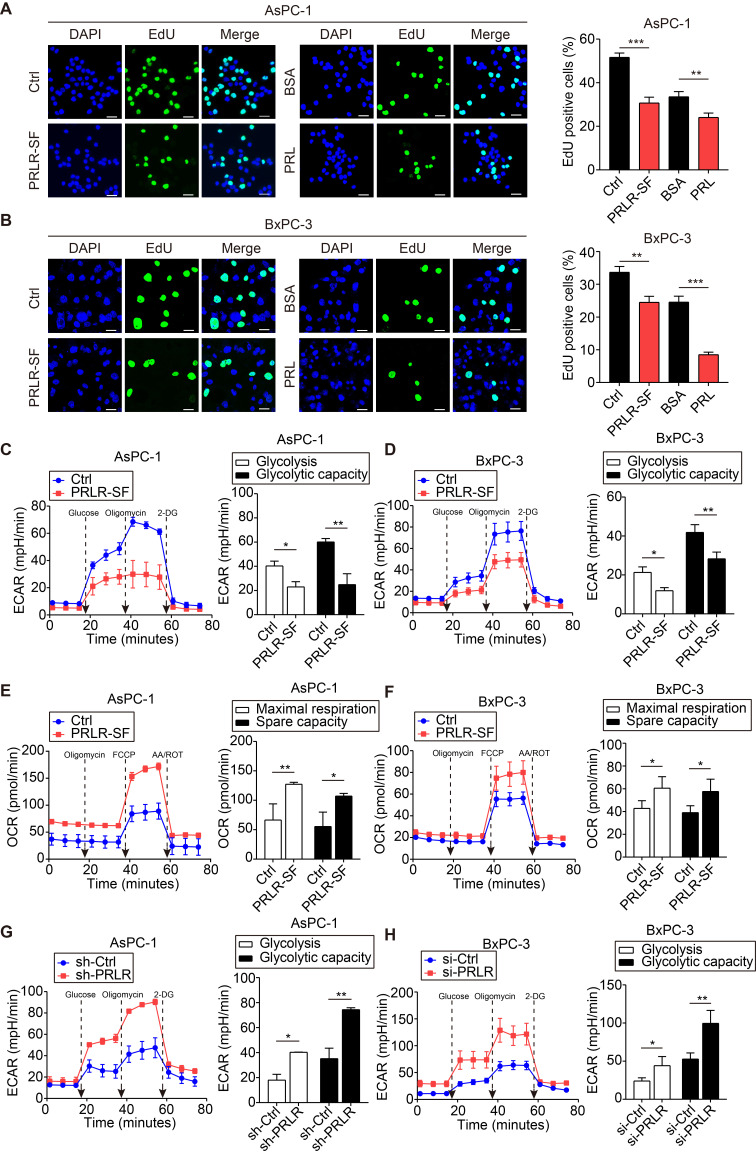
** PRLR-SF inhibits DNA synthesis and glycolysis of PDAC cells.** (**A**, **B**) Representative images of DNA synthesis, detected by EdU incorporation (green) in AsPC-1 cells (**A**) or BxPC-3 cells (**B**) incubated with 0.1%BSA or prolactin (PRL). DAPI staining indicates nuclei (blue). Histograms show percentages of EdU-positive cells (n = 3). (**C**, **D**) The effects of PRLR-SF overexpression on Extracellular acidification rate (ECAR) in AsPC-1 (**C**) and BxPC-3 (**D**) cells. Glycolysis and glycolytic capacity are calculated. (**E**, **F**) The effects of PRLR-SF overexpression on Oxygen consumption rate (OCR) in AsPC-1 (**D**) and BxPC-3 (**F**) cells. Maximal respiration and spare capacity are calculated. (**G**, **H**) The effects of knockdown PRLR on ECAR in AsPC-1 (**G**) and BxPC-3 (**H**) cells. Glycolysis and glycolytic capacity are calculated. *P* values were determined by unpaired t-test. *P < 0.05, **P < 0.01, ***P < 0.001.

We investigated whether the biosynthesis of molecules in the PPP was affected by prolactin. G6PD is a rate-limiting enzyme that regulates the oxidative PPP, generating ribose-5-phosphate (R5P) for the *de novo* synthesis of nucleotides and producing NADPH, which neutralizes reactive oxygen species 34. We used [U^13^C]-labeled glucose to trace R5P and downstream nucleotides generated from PPP fluxes. M+5-labeled R5P was decreased in the PDAC cells that overexpressed PRLR-SF compared with the expression level in the control cells (Figure 2J). When AsPC-1 cells were incubated with prolactin, the R5P level was decreased, but when a blocking antibody was added, the amount of U-^13^C-incorporated R5P was significantly increased (Figure 2J). Overexpression of PRLR-SF in PDAC cells and incubation with prolactin increased the ratio of NADP^+^ to NADPH (Figure 2K). PRLR-SF overexpression and incubation of PDAC cells with prolactin (PRL) inhibited DNA synthesis, as measured by EdU incorporation (Figure 3A, B). These data suggest that PRLR-SF decreases PPP biosynthesis and DNA synthesis. The inhibition of G6PD and TKT (6-AN and OT) and siRNA against G6PD blocked the promotion of sh-PRLR during cell proliferation (Figure S4G-I). The overexpression of G6PD reversed the inhibition of cell proliferation induced by PRLR-SF (Figure 2 L); the overexpression of TKT did not have an effect (data not shown).

TKT is a metabolic enzyme involved in the nonoxidative branch of the PPP, connecting it with glycolysis. It is required for cancer growth 35, 36. Reduced TKT results in a switch from glucose metabolism via glycolysis to oxidative phosphorylation 37. We showed that an inhibitor of TKT (OT) restricted glycolysis in PDAC cells (Figure S4J, K). Furthermore, the overexpression of PRLR-SF reduced the extracellular acidification rate (ECAR) (Figure 3C, D) and increased the oxygen consumption rate (OCR) (Figure 3E, F). PRLR-SFs knockdown by shRNA and siRNA led to the opposite results (Figure 3G, H). These data indicate that PRLR-SF overexpression regulates glucose metabolism by inhibiting glycolysis and promoting oxidative phosphorylation in PDAC cells.

### PRLR-SF activates the Hippo pathway in PDAC

We next explored how PRLR signaling is activated in PDAC cells. We found that downstream signals of PRLR-LF, including STAT5, AKT, and ERK1/2, were not obviously phosphorylated in BxPC-3 and AsPC-1 cells overexpressing PRLR-SF (Figure S5A, EGF treatment was designed as a positive control). The results showed that these inhibitors suppressed both AsPC-1-Ctrl and AsPC-1-sh-PRLR cell growth; however, the inhibitors did not diminish the growth difference between these two cell lines (Figure S5B-E). These results suggest that the activation of PRLR-LF is not a major factor in these PDAC cells, and PRLR-SF might stimulate pathways that differ from those stimulated by PRLR-LF to regulate cell proliferation.

**Figure 4:**
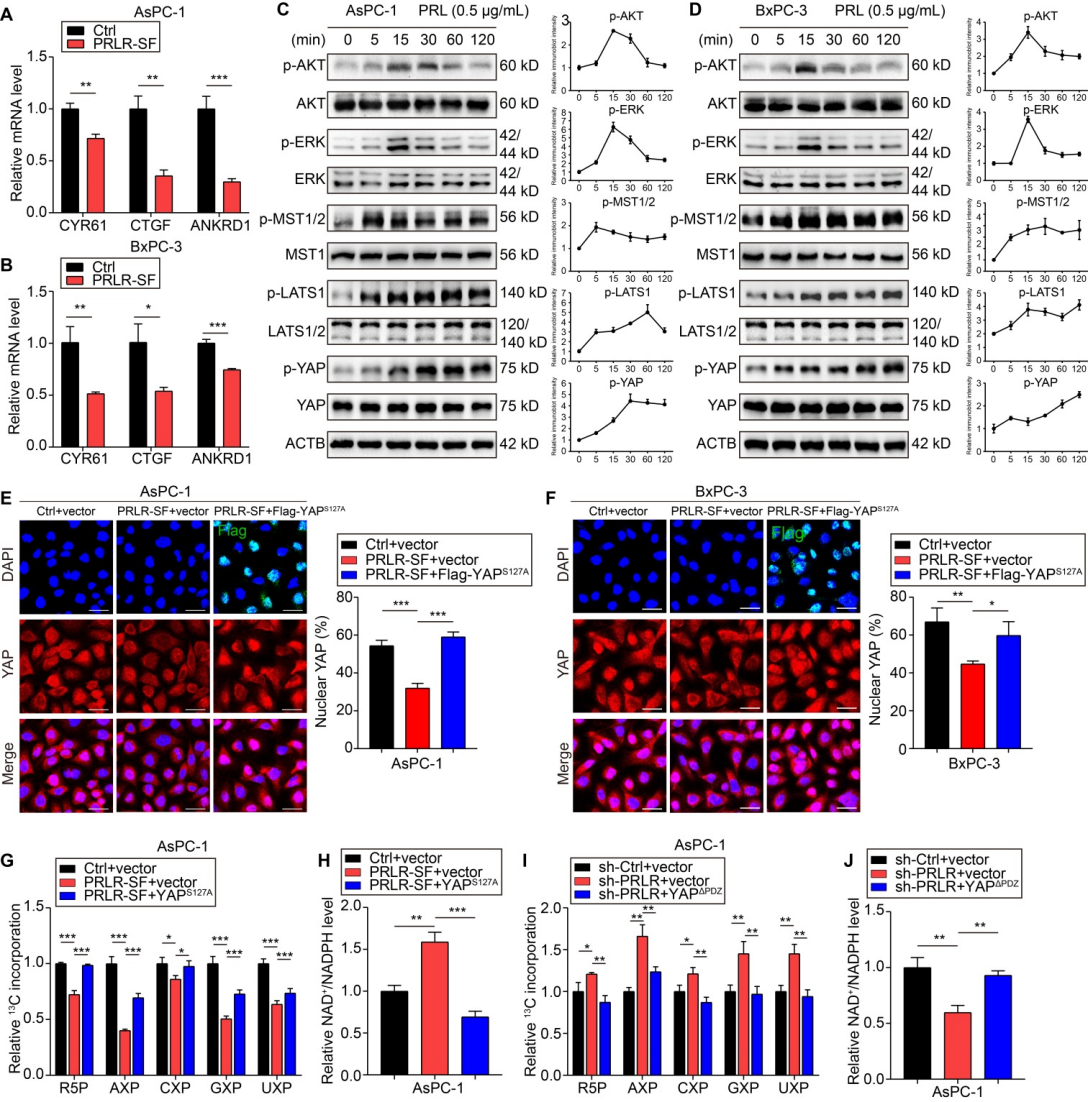
** Hippo signaling pathway is activated by PRLR-SF in PDAC cells.** (**A**,** B**) Relative levels of *CYR61*, *CTGF*, and *ANKRD1* mRNAs in control (Ctrl) AsPC-1 cells (**A**) and BxPC-3 cells (**B**) vs those that overexpress PRLR-SF (n = 3). (**C, D**) Immunoblots showing levels and phosphorylation (p) of AKT, ERK1/2, and proteins in the Hippo signaling pathway in AsPC-1 cells and BxPC-3 cells incubated with 500 ng/mL prolactin (PRL) for 0-120 mins in serum-free media. (**E**, **F**) Representative immunofluorescence images of AsPC-1 cells (**E**) or BxPC-3 cells (**F**) infected with Lenti-control (Ctrl) or Lenti-PRLR-SF, and transfected transiently with empty vectors (vector) or vectors that overexpress Flag-YAP^S127A^. Flag labeled in green, nuclei labeled in blue, YAP labeled in red, and combined images below. Scale bar = 25 μm. Quantitative histograms show percentages of nuclear YAP in each cell type (n = 3). (**G**, **H**) Histograms of relative incorporation of ^13^C-labeled R-5-P, AXP, CXP, GXP, and UXP (X = M, D and T) (**G**) or ratio of NADP^+^ to NADPH (**H**) in AsPC-1 cells infected with Lenti- Ctrl or Lenti-PRLR-SF, transfected with empty vector vs vector expressing YAP^S127A^. (**I**, **J**) Histograms of relative incorporation of ^13^C-labeled R-5-P, AXP, CXP, GXP, and UXP (**I**) or ratio of NADP^+^ to NADPH (**J**) in AsPC-1 cells infected with Lenti-sh-Ctrl or Lenti-sh-PRLR, transfected with empty vector vs vector expressing YAP^ΔPDZ^.* P* values were determined by unpaired t test. *P < 0.05, **P < 0.01, ***P < 0.001.

In the analysis of the cDNA microarray data, we found that the expression of genes regulated by the transcriptional coactivators YAP (yes-associated protein)/TAZ with a PDZ-binding motif (WW domain-containing transcription regulator 1, WWTR1, also called TAZ) was altered in PRLR-SF-overexpressing cells. PRLR-SF overexpression downregulated the transcription of CRY61, CTGF, and ANKRD1, which are regulated by YAP (Figure 4A, B). YAP and TAZ are transcriptional regulators in the Hippo pathway, which controls cell proliferation and apoptosis 38, 39. The components of the Hippo pathway, MST1, LATS1/2, and YAP, were persistently phosphorylated in the PDAC cells incubated with prolactin compared to those in cells with transient phosphorylation of AKT and ERK1/2 (Figure 4C, D, Figure S5F, G). To further confirm that the downstream signaling triggered by PRLR-SF is different from PRLR-LF, we investigated Hippo pathway activation in PANC-1 cells, which mainly express PRLR-LF rather than PRLR-SF. The results showed that in response to prolactin treatment, ERK/AKT was strongly phosphorylated, and Hippo activation was almost negligible. However, with PRLR-SF overexpression in PANC-1 cells, the prominent activation of ERK/AKT was diminished. Instead, Hippo pathway phosphorylation was intensively promoted (Figure S5H, I), and YAP nuclear localization was decreased (Figure S6A, B). These data provide evidence that PRLR-SF activation induced other signaling pathways that differed from PRLR-LF. IHC staining of tumor tissue generated from subcutaneously transplanted cells showed similar results (Figure S6C). These results indicate that PRLR-LF activation is not dominant in these PDAC cells and that PRLR-SF signaling may be distinct from that of PRLR-LF, and it seems to activate the Hippo pathway in these PDAC cells.

When phosphorylated, YAP forms a complex with TAZ and remains in the cytoplasm instead of being translocated to the nucleus to initiate transcription. We found that PDAC cells that overexpressed PRLR-SF had a significant reduction in the nuclear localization of YAP (Figure 4E, F). However, in the cells that expressed a mutant form of YAP (YAP S127A), which is not phosphorylated at serine 127, the expression of PRLR-SF did not maintain the YAP level in the cytoplasm (Figure 4E, F). Moreover, si-PRLR-mediated YAP nuclear localization was reversed by the YAP cytoplasmic localization mutant (YAPΔPDZ) (Figure S6D-G).

**Figure 5:**
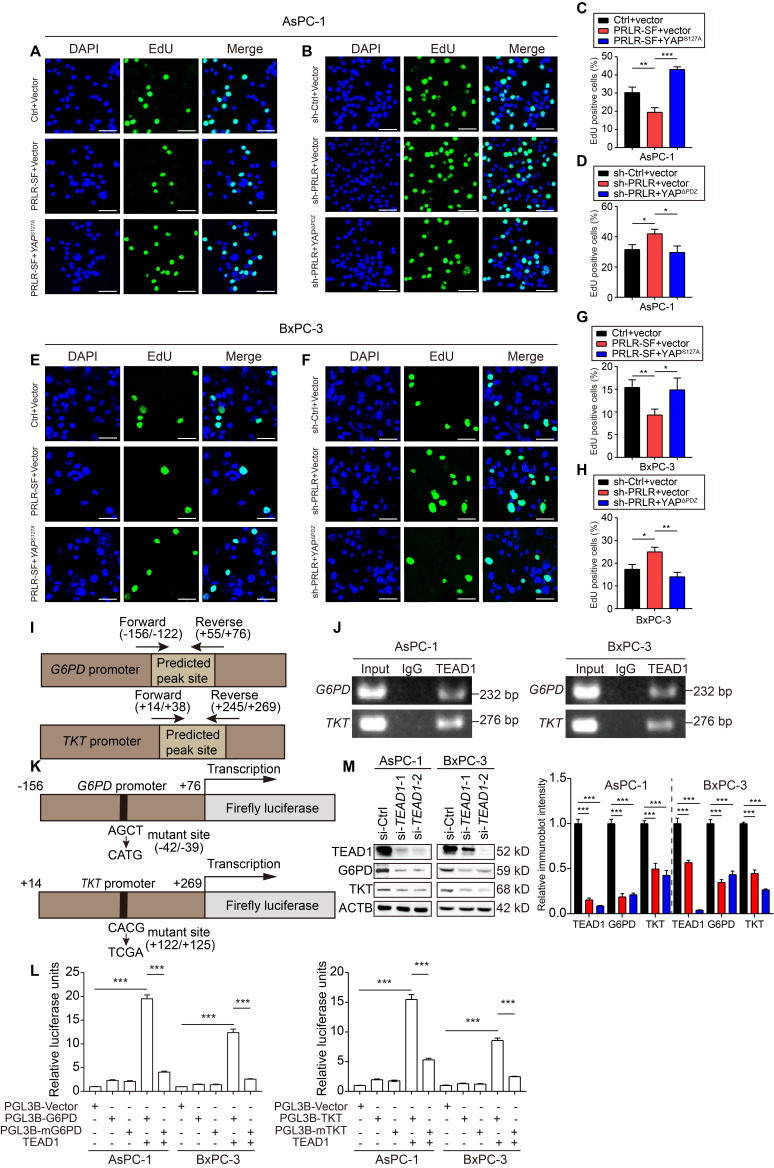
** PRLR-SF inhibits DNA synthesis through Hippo signaling pathway via directly targeting G6PD and TKT in PDAC cells.** (**A**-**D**) Representative images of DNA synthesis, detected by EdU incorporation (green) in AsPC-1 cells infected with Lenti- Ctrl or Lenti-PRLR-SF, transfected with empty vector vs vector expressing YAP^S127A^ (**A**,** C**), or infected with Lenti-sh-Ctrl or Lenti-sh-PRLR, transfected with empty vector vs vector expressing YAP^ΔPDZ^ (**B**,** D**). DAPI staining (blue) shows nuclei. Histograms show percentages of EdU-positive cells (n = 3). (**E**-**H**) Representative images of DNA synthesis, detected by EdU incorporation (green) in BxPC-3 cells infected with Lenti- Ctrl or Lenti-PRLR-SF, transfected with empty vector vs vector expressing YAP^S127A^ (**E**,** G**), or infected with Lenti-sh-Ctrl or Lenti-sh-PRLR, transfected with empty vector vs vector expressing YAP^ΔPDZ^ (**F**,** H**). DAPI staining (blue) shows nuclei. Histograms show percentages of EdU-positive cells (n = 3). *P* values were determined by unpaired *t* test. (**I**) Predicted peak sites of TEAD1 binding to the *G6PD* or *TKT* promoter. (**J**) Agarose gel electrophoretic images of CHIP-PCR assays demonstrate binding of TEAD1 to promoter regions of *G6PD* and *TKT* in AsPC-1 cells (left) and BxPC-3 cells (right). (**K**) Sites of mutations in promoters of *G6PD* and *TKT* in luciferase reporter constructs. (**L**) Relative luciferase activities in AsPC-1 and BxPC-3 cells transfected with only PGL3B vector, vector expressing G6PD (left) or TKT (right), mutant (m) G6PD or TKT, with or without expression of TEAD1 (n = 3). *P* values were determined by unpaired t test. ***P < 0.001. (**M**) Immunoblot analyses for the effects of interfering *TEAD1* on protein expression of G6PD and TKT using AsPC-1 cells (left) and BxPC-3 cells (right). *P < 0.05, **P < 0.01, ***P < 0.001.

The levels of R5P and nucleotides (adenine nucleotides (AXP), uracil nucleotides (UXP), guanine nucleotides (GXP), and cytosine nucleotides (CXP)) decreased when the PDAC cells overexpressed PRLR-SF but not when the cells also expressed YAP S127A (Figure 4G). The ratio of NADP^+^ to NADPH increased when the PDAC cells overexpressed PRLR-SF but not when the cells coexpressed YAP S127A (Figure 4H). In contrast, the R5P and nucleotide levels that had been increased by sh-PRLR were abolished by the YAP cytoplasmic localized mutant (YAPΔPDZ) (Figure 4I). The NADP^+^: NADPH ratio that had been reduced by sh-PRLR was rescued by YAPΔPDZ in the PDAC cells (Figure 4J). In addition, both YAP mutants rescued DNA synthesis alterations caused by PRLR overexpression or PRLR knockdown (Figure 5A-H). The downregulated glycolytic capacity induced by PRLR-SF was also reversed by the YAP mutant (Figure S6H, I). Collectively, these data indicate that PRLR regulates PPP biosynthesis and glucose metabolism through the Hippo pathway in these PDAC cells.

### TEAD1 directly regulates the transcription of *G6PD* and *TKT*

YAP is a transcriptional coactivator of TEAD family members, which share a highly conserved DNA-binding domain. To investigate whether *G6PD* and *TKT* are target genes of TEAD family proteins, we performed bioinformatics analysis using the chromatin immunoprecipitation (ChIP) sequence database. We found that TEAD1 with *G6PD* and *TKT* showed a prominent binding peak. To verify the direct binding of TEAD1 to the promoters of *G6PD* and *TKT*, we performed ChIP-PCR assays. The results showed that TEAD1 was directly bound to the promoter regions of the *G6PD* and *TKT* genes in PDAC cells (Figure 5I, J). We cloned the promoter regions of *G6PD* and *TKT* that contained the TEAD1-binding site, or mutant sequences, into a luciferase reporter gene construct. Transcription of the luciferase gene under the control of the wild-type *G6PD* or *TKT* promoter was significantly activated by TEAD1, whereas transcription under the control of the mutated promoter was greatly reduced, indicating that these promoters have effective TEAD1-binding sites (Figure 5K, L). When PDAC cells were transfected with siRNAs targeting TEAD1, the protein levels of G6PD and TKT were downregulated (Figure 5 M, Figure S7A). These results indicate that Hippo signaling affects the PPP via the direct transcriptional regulation of *G6PD* and *TKT* by TEAD1 proteins in PDAC cells.

### PRLR-SF activates the Hippo pathway through physical interactions with NEK9

**Figure 6:**
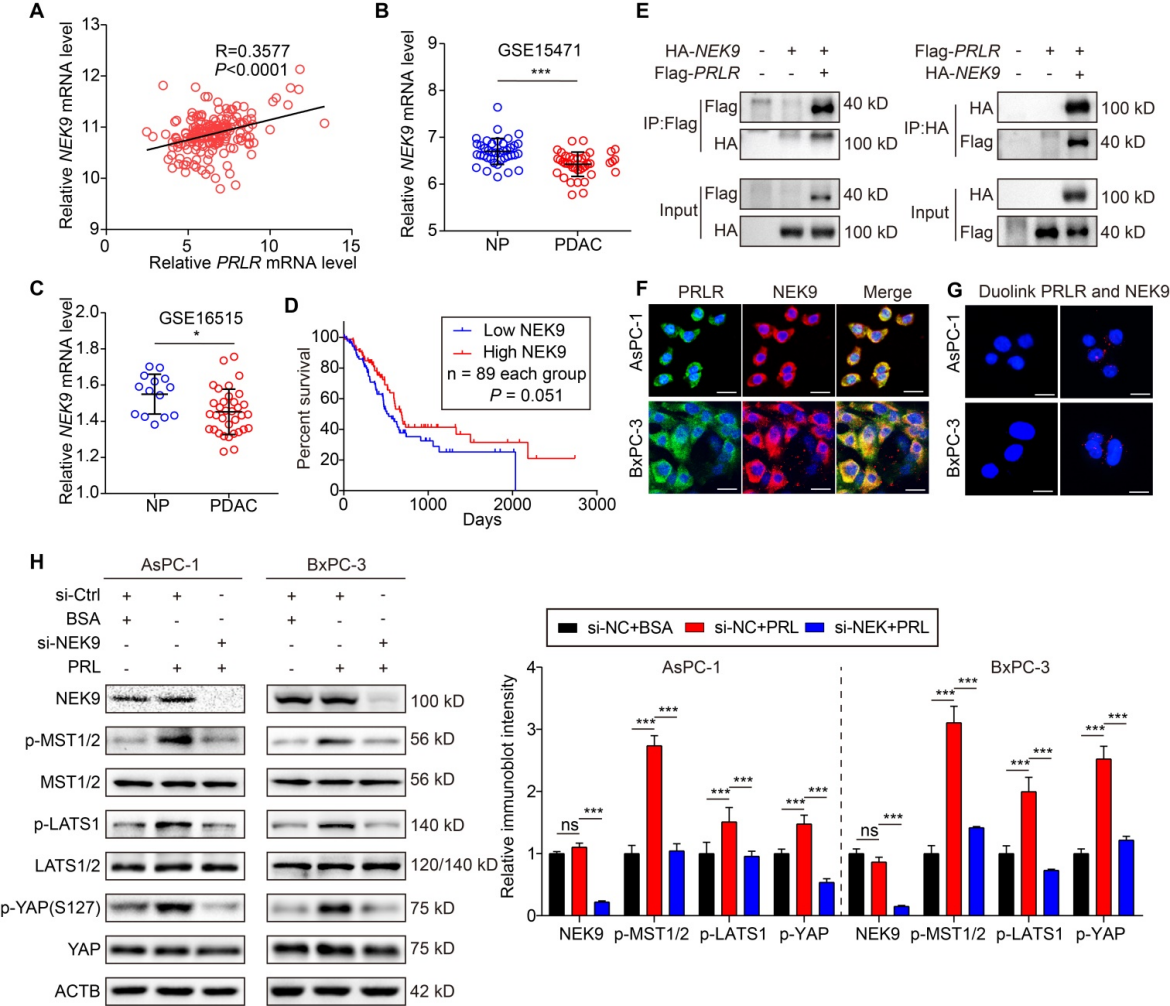
** PRLR activates Hippo pathway via interaction with NEK9.** (**A**) Linear regression analysis for *PRLR* and *NEK9* mRNA level of PDAC patients based on TCGA database. n = 182. Pearson correlation coefficients (R) are shown. (**B**, **C**) Relative *NEK9* mRNA level of paired non-PDAC ('NP') and PDAC tissues in GSE15471 (**B**) and GSE16515 (**C**). *P* values were determined by paired t test. (**D**) Kaplan-Meier survival analysis for PDAC patients divided into low or high *NEK9* mRNA level group based on TCGA. *P* value was calculated with log-rank (Mantel-Cox) test. (**E**) Immunoblot analyses for co-immunoprecipitation of NEK9 and PRLR using AsPC-1 cells co-transfected with HA-tagged NEK9 and Flag-tagged PRLR. (**F**) Immunofluorescence for co-localization of PRLR (green) and NEK9 (red) in AsPC-1 cells (top) and BxPC-3 (bottom) cells. (**G**) Immunofluorescence for the interactions (red dots) of PRLR and NEK9 detected by *in situ* proximity ligation assays using AsPC-1 cells (top) and BxPC-3 cells (bottom). (**H**) Immunoblot analyses for the effects of interfering NEK9 on Hippo signaling pathway using AsPC-1 (left) and BxPC-3 (right) cells stimulated with PRL (0.5 μg/ml). Scale bar: 10 μm. *P < 0.05, ***P < 0.001.

To determine the underlying mechanisms by which PRLR-SF activates the Hippo pathway, we sought to discover the intermediator between PRLR and the Hippo pathway. Previous works reported that PRLR interacts with NIMA-related kinase 3 (NEK3), a member of the NimA (never in mitosis A) family of serine/threonine protein kinases 40. NEK2A, another member of the NEK family, was reported to directly interact with the Hippo pathway component Mst2 41. Thus, we speculated that a NEK family member might be an intermediator between PRLR-SF and the Hippo pathway in PDAC cells. By analyzing the TCGA database, we found that among the 11 members of the NEK family, the mRNA expression of NEK9 was the most closely correlated with the expression of PRLR (Figure 6A). Furthermore, we analyzed two GEO datasets (GSE15471 and GSE16515) and found that NEK9 expression was downregulated in PDAC tissues (Figure 6B, C). Low NEK9 mRNA levels were correlated with poor overall survival according to the TCGA data set analysis (Figure 6D).

We next determined whether NEK9 directly interacts with PRLR-SF in PDAC cells. Coimmunoprecipitation of exogenous PRLR-SF and NEK9 revealed that NEK9 was present in the anti-PRLR precipitates of PDAC cells (Figure 6E). Next, by proximity ligation assays and immunofluorescent colocalization assays, we confirmed a physical interaction between PRLR and NEK9 (Figure 6F, G). To further demonstrate that NEK9 serves as an intermediary signaling molecule between PRLR and the Hippo pathway, NEK9 was silenced by siRNA in PDAC cells. As expected, the knockdown of NEK9 greatly diminished the PRL-induced activation of MST/LATS/YAP (Figure 6H, Figure S7B). Nuclear YAP was increased by PRL, and the increase was reversed by siNEK9 (Figure S7C-F). These data provide evidence that PRLR-SF activates the Hippo pathway through physical interactions with NEK9 in PDAC cells. Together, these findings establish that PRLR-SF inhibits PPP flux induced by the NEK9-Hippo-G6PD/TKT axis.

### The PRLR level in PDAC tissue correlates with patient outcomes

**Figure 7:**
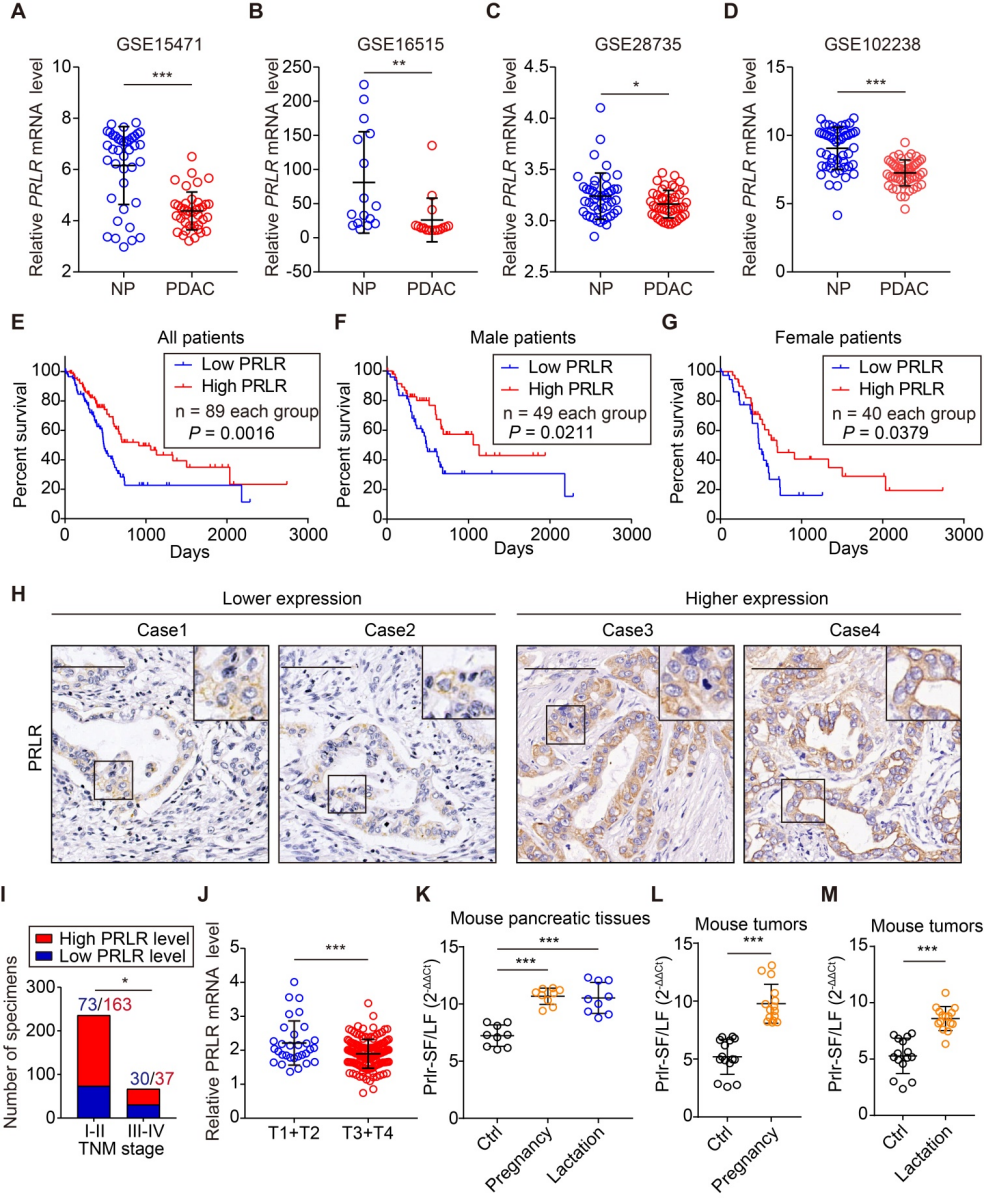
** PRLR level in PDAC tissue correlates with clinicopathological findings.** (**A**-**D**) Relative *PRLR* mRNA level of paired NP and PDAC tissues in GSE15471 (**A**), GSE16515 (**B**), GSE28735 (**C**) and GSE102238 (**D**). *P* values were determined by paired two-tailed t-test. (**E**) Kaplan-Meier survival analysis for all PDAC patients divided into low and high *PRLR* mRNA level group based on TCGA data. *P* value was calculated with log-rank (Mantel-Cox) test. (**F**, **G**) Kaplan-Meier survival analysis for male PDAC patients (**F**) or female PDAC patients (**G**) divided into low and high *PRLR* mRNA level group based on TCGA data. *P* value was calculated with log-rank (Mantel-Cox) test. (**H**) Representative immunohistochemical staining images for showing lower (left) and higher (right) PRLR expression levels using patient-derived PDAC tissues. Scale bar: 100μm. (**I**) Correlation analyses for PRLR expression with TNM stage of patients with PDAC from Ren Ji cohort. *P* value was determined by χ2 test. (**J**) Relative *PRLR* mRNA level for T1+T2 stages and T3+T4 stages of patients based on TCGA data. *P* values were determined by unpaired two-tailed t-test. (**K**) Ratio of Prlr-SF: Prlr-LF in pancreas of non-pregnant mice (Ctrl), pregnant mice (pregnancy), and lactating mice (lactation), based on 3 samples from each group and 3 replicates of each sample. *P* values were determined using the unpaired 2-tailed test. (**L**,** M**) Ratios of PRLR-SF: PRLR-LF in subcutaneous xenografts grown from Panc02 cells in non-pregnant (Ctrl) and pregnant mice (**L**) and in non-lactating (Ctrl) vs lactating mice (**M**). Five samples from each group and 3 replicates were analyzed for each sample. *P* values were determined by unpaired 2-tailed test. *P < 0.05, **P < 0.01, ***P < 0.001.

We further investigated the clinical significance of PRLR expression. By analyzing the GEO data sets (GSE15471, GSE16515, GSE28735 and GSE102238), we found that PRLR and PRL expression was downregulated in PDAC patient tissue compared to the corresponding paracancerous tissue (Figure 7A-D, Figure S8A-C). A similar result was obtained from the analysis of combined TCGA and GTEx data (Figure S8D). Analysis of the TCGA and GTEx databases of pancancers revealed that the mRNA levels of PRL and PRLR were downregulated in most types of tumors (Figure S8E, F).

We analyzed the levels of *PRLR* mRNA in tumor tissues using the TCGA database and found a correlation between high levels of *PRLR* mRNA and longer survival times for patients with PDAC (Figure 7E). The survival analysis of male and female patients showed similar results (Figure 7F, G). We performed a large-scale IHC analysis in a tissue microarray containing 300 pathologist-certified and clinically annotated PDAC specimens (Figure 7H). The correlation between PRLR levels and clinical parameters was assessed. The results demonstrated that the PRLR expression level was negatively correlated with the TNM stage of the PDAC patient tissues (Figure 7I). A similar result was obtained from TCGA database analysis (Figure 7J). These data indicate that PRLR might slow PDAC progression.

We found that PRLR-SF, but not PRLR-LF, was predominantly expressed in pancreatic tissues in female wild-type mice (Figure 7K, Figure 1C). Many epidemiological studies have investigated the inverse association between pregnancy or lactation and cancer risk 42-45. The secretion of prolactin remarkably increases during pregnancy and lactation 46, implying that PRL/PRLR might contribute to the correlation between cancer development and pregnancy/lactation. We investigated whether pregnancy and lactation affect the expression of PRLR isoforms in the pancreas and pancreatic tumors of mice. The pancreatic tissues from 12-week-old pregnant and lactating WT mice had increased ratios of PRLR-SF: PRLR-LF (Figure 7K). Pregnancy and lactation also increased the ratio of PRLR-SF:PRLR-LF in the subcutaneous xenograft PDAC tumors compared with the nonpregnant and nonlactating mice (Figure 7L-M). By analyzing the weight of the tumors from pregnant and lactating mice, we found that tumors in pregnant/lactating mice were smaller than those in the control group (Figure S9A, B). Together with the suppressive role of PRLR-SF on PDAC cell growth, these results suggest that alteration of the PRLR-SF: PRLR-LF ratio induced by pregnancy and lactation might contribute to a reduced cancer risk in women.

## Discussion

PRLR isoforms have different signaling properties. PRLR-SF is not phosphorylated on a tyrosine, which prevents it from interacting directly with SH2-containing proteins such as STAT factors 1, 17. We investigated the effects of prolactin signaling via its receptor (PRLR) in PDAC cells. We discovered that PRLR-SF activates a signaling pathway distinct from that of PRLR-LF in PDAC cells. PRLR-SF inhibits PDAC cell proliferation, colony formation, and the development of xenograft tumors in mice, indicating that this isoform somehow prevents tumor progression.

Metabolic reprogramming is a core hallmark of cancer, but it remains poorly defined in pancreatic cancer 47. Studies on rodents have reported that prolactin regulates glucose metabolism through its effects on pancreatic β-cell mass and insulin production 48. In addition, prolactin might also affect energy homeostasis by modulating lipid metabolism 49. Direct regulation of PRLR in glucose metabolism pathway has rarely been reported. Cancer cells frequently have metabolic alterations that endow them with proliferative advantages 50, 51. In cancer cells, the PPP not only generates pentose phosphates to supply a high rate of nucleic acid synthesis but also provides NADPH, which is required for the synthesis of fatty acids and cell survival under stress conditions 52. Although the PPP has been targeted for cancer therapy 53, little is known about the PPP in PDAC. We showed that PRLR-SF regulates the PPP by reducing the expression of two rate-limiting enzymes in pentose phosphate metabolism, G6PD and TKT, linking prolactin signaling to metabolic reprogramming in PDAC.

Our findings indicate that Hippo signaling is the intermediator between PRLR-SF and pentose phosphate metabolism. The Hippo pathway was found to be a conserved tumor suppressor pathway restricting cell proliferation and promoting apoptosis. With its nuclear effector YAP, Hippo regulates organ size and cancer formation. As many cancers are marked by unchecked cell division, this signaling pathway has been increasingly found to be significant in human cancers 39. Although many modulators of Hippo activity have been identified, little is known about Hippo target genes that mediate growth effects. Several glucose metabolism genes, including glucose transporter 3 GLUT3 54, 55, hexokinase 2 (HK2) and phosphofructokinase B3 (PFKB3), have been confirmed to be indirect transcriptional targets of YAP 56, 57. In our study, we confirmed the direct transcriptional regulation of G6PD and TKT by TEAD1, a downstream transcription factor in the Hippo pathway, in PDAC cells. Therefore, these data provide a link between the Hippo pathway and glucose metabolism that has exciting implications for cancer prevention and expanding the understanding of the biological characteristics of the Hippo pathway in cancer.

NEK9 is known to play a role in spindle assembly and in the control of centrosome separation, but the consequences of NEK9 targeting in cancer cells remain to be elucidated. NEK9 correlates with a worse overall survival of melanoma patients 58 and is upregulated in meningioma 59. However, a low level of NEK9 mRNA is common in triple-negative breast cancers and is associated with poor overall survival and distant metastasis-free survival 60. With the TCGA and GEO data set analyses, we found that NEK9 expression is downregulated and that low levels of NEK9 predict a poor outcome for PDAC patients. We also verified that NEK9 interacts with PRLR-SF and is involved in the Hippo activation induced by PRLR-SF in PDAC cells. These data provide new evidence for the biological features and downstream signals of NEK9 in tumor development, suggesting that, in addition to its function in centrosome separation, NEK9 also regulates cell proliferation via the Hippo pathway.

PRLR-SF inhibits the expression of PRLR-LF through the accelerated degradation of PRLR-LF mRNA 8, 61. Cells that expressed transgenic PRLR-SF expressed lower levels of PRLR-LF. When the ratio of PRLR-SF to PRLR-LF reached 1:1, the expression of PRLR-LF was decreased by 90% 19. PRLR-SF not only downregulates the expression of PRLR-LF but also acts as a dominant negative factor of the LF by LF-SF heterodimerization 8, 62. Different expression ratios might result in the inhibition or promotion of tumor progression. An NIH study showed that the ratio of PRLR-SF to PRLR-LF was significantly decreased in 76% of breast cancer patients. Because SFs act as dominant negative regulators of the stimulatory actions of LF *in vitro*, their relatively reduced expression in cancer could cause gradations in LF stimulatory function and contribute to breast tumor progression 63. Our study reveals that, in addition to its role in eliminating PRLR-LF, PRLR-SF functions independently to trigger a specific pathway that suppresses the proliferation of PDAC cells.

Lactation was recently reported to be associated with a 24% lower risk of invasive ovarian cancer, particularly high-grade serous and endometrioid cancers 64. Lactation also reduces a woman's risk of breast cancer 65, 66. Moreover, retrospective cohort studies from 2003 through 2016 showed that infertile women have an overall higher risk of developing cancer than fertile women. The risks of uterine cancer, ovarian cancer, lung cancer, thyroid cancer, liver and gallbladder cancer, and leukemia are higher in infertile women than in fertile women 42. Additionally, women who are pregnant at later ages (> 26 years old) have a lower risk of noncardia gastric cancers 43.

All these evidences imply that pregnancy and lactation might be modifiable factors that significantly decrease the cancer risk. During pregnancy, prolactin is produced in high quantities by the maternal and fetal pituitary and the decidua 6. Prolactin secretion increases gradually, beginning at 6-8 weeks gestation and is maintained at high levels until term 46. The level of placental lactogen, a ligand of the prolactin receptor (PRLR), is also increased to a greater extent. By 30 weeks of pregnancy, the levels of placental lactogen exceed the levels of prolactin by 10-fold 6. In our study, we showed that pregnancy and lactation increased the ratio of PRLR-SF:PRLR-LF in pancreatic tissues from wild-type mice and tumors from mice with subcutaneously transplanted PDAC cells. The increase in PRLR-SF during pregnancy and lactation might help reduce the risk of PDAC and other cancers by dual functions: trigging the Hippo-PPP pathway and diminishing PRLR-LF effects. Further studies are needed to determine how pregnancy and lactation induce PRLR-SF: PRLR-LF ratio alterations in the pancreas and PDAC.

In summary, we made the interesting observation that PRLR-SF signals induced via the NEK9-Hippo pathway reduce the expression of G6PD and TKT in the PPP and thereby reduce pancreatic tumor progression. Hence, targeting PRLR-SF-mediated metabolic reprogramming pathways may be a potential therapeutic strategy for PDAC prevention and treatment.

## Supplementary Material

Supplementary figures and tables.

Supplementary cDNA microarray.

Supplementary patient information.

## Figures and Tables

**Figure 8:**
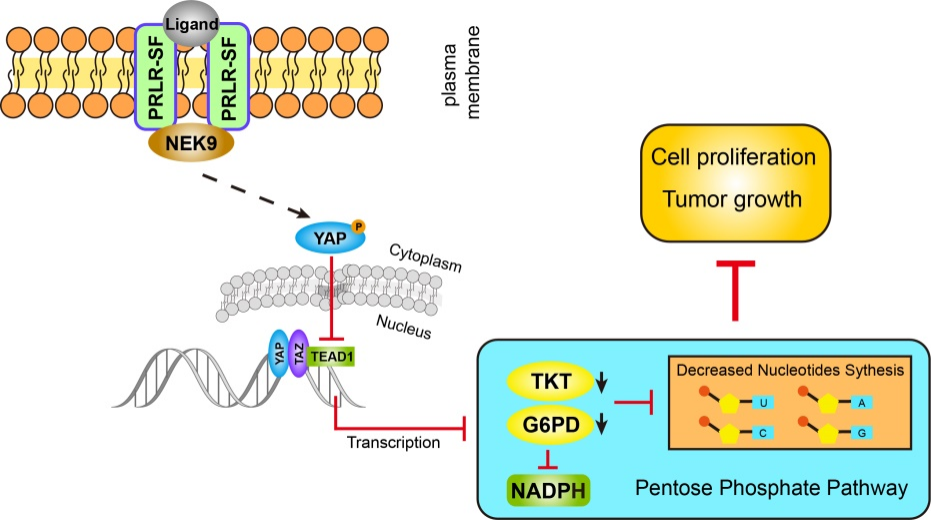
PRLR-SF inhibits pentose phosphate pathway through NEK9-YAP/TEAD1-G6PD/TKT axis in PDAC cells.

## Abbreviations

CHIP: chromatin immunoprecipitation
ECAR: extracellular acidification rate
GEO: gene expression omnibus
GTEx: genotype-tissue expression
KPC: Kras^G12D/+^/Trp53^R172H^/+/Pdx1-Cre
PRLR-LF: long isoform of prolactin receptor
NEK9: NIMA related kinase 9
OCR: oxygen consumption rate
PDAC: pancreatic ductal adenocarcinoma
PRL: prolactin
PRLR: prolactin receptor
R5P: ribose-5-phosphate
PRLR-SF: short isoform of prolactin receptor
PPP: pentose phosphate pathway
G6PD: Glucose-6-phosphate dehydrogenase
TKT: transketolase
TCGA: the cancer genome atlas
ORF: open reading frame
