# Supplementary material for: The short isoform of PRLR suppresses the pentose phosphate pathway and nucleotide synthesis through the NEK9-Hippo axis in pancreatic cancer
Source: Theranostics. 2021 Feb 6;11(8):3898–915. doi: 10.7150/thno.51712 (PMC7914341; doi:10.7150/thno.51712)
Supplement: Supplementary file 1 — Supplementary figures and tables. [file thnov11p3898s1.pdf]

Materials and Methods

Cell lines and cell culture. Human pancreatic ductal cell line hTERT-HPNE, PDAC cell lines SW-1990, AsPC-1, PANC-1, Capan-1, CFPAC-1, BxPC-3, Patu8988 and mouse PDAC cell line FC1199 were all preserved in Shanghai Cancer Institute, Ren Ji Hospital, School of Medicine, Shanghai Jiao Tong University. All cell lines have been performed STR identification and excluded endogenic contamination. Cells were cultured at 37 °C in a humidified incubator under 5% CO_2 condition, with indicated medium containing 10% (v/v) fetal bovine serum (FBS) and 1% (v/v) antibiotics, according to American Type Culture Collection (ATCC, Manassas, VA) protocols.

Animal studies. Animal experiments were approved by Institutional Animal Care and Use Committee of East China Normal University. Mice were manipulated and housed according to the criteria outlined in the Guide for the Care and Use of Laboratory Animals prepared by the National Academy of Sciences and published by the National Institutes of Health.

Pregnant and lactational mice model. The animal models used in detecting PRLR-SF/LF ratio were C57BL/6 female mice. For obtaining normal pancreatic tissues, pregnant mice or lactational mice were sacrificed at day 20 of pregnancy or lactation. All mice including control mice were sacrificed at the same weeks old.

For obtaining subcutaneous mouse-derived xenograft, a total of 2×10^6 FC1199 cells were injected subcutaneously in the right back of the mouse once it was pregnant or started breastfeeding. All mice including control mice were sacrificed after injection for 20 days at the same age.

Subcutaneous and orthotopic human-derived xenograft model. Athymic female nu/nu mice aged from 6 to 8 weeks were used in these experiments. For subcutaneous xenograft model in PRL stimulation study, a total of 5×10^6 AsPC-1 cells in 150 μl PBS were injected subcutaneously in the right back of the mouse. When tumors were visible, these mice were divided into two groups (Vehicle and PRL) randomly. Tumors of mice in vehicle or PRL group were injected with 50 μl 0.1% (m/v) BSA or reconstitute prolactin (0.5 μg/ml, diluted in 0.1% BSA, Peprotech, 100-07) respectively every day lasting for 10 days. Then mice were sacrificed, and tumors were isolated and weighted.

For orthotopic xenograft model in genetic overexpression study, 10 nude mice were averagely divided into two groups randomly. A total of 2×10^6 Ctrl or PRLR-SF AsPC-1 cells or BxPC-3 cells in 25 μL PBS were injected into the head or body of pancreas. After 4 weeks, mice were sacrificed, and the whole pancreas coherent with tumor and spleen was obtained and photographed, then tumor was isolated and weighted.

For survival analysis of nude mice orthotopically implanted with AsPC-1 cells, the treatments were same as orthotopic xenograft model. Survival days were recorded once the mouse was dead.

Western blotting. Cells were lysed with RIPA (50 mM Tris (pH 7.4), 150 mM NaCl, 1% Triton X-100, 1% sodium deoxycholate， 0.1% SDS) containing completed protease inhibitor (complete, Roche) or phosphatase inhibitor (PhosSTOP, Roche) and collected for centrifugation . The supernatant total protein concentration was measured with BCA Protein Assay Kit (Pierce Biotechnology, Waltham, MA). After denaturation with loading buffer, the cell lysates were separated with 8%-12% sodium dodecyl sulfate (SDS) –polyacrylamide gel electrophoresis (PAGE) gel electrophoresis and transferred to a nitrocellulose membrane. The membrane was blocked with 5% (m/v) skim milk and incubated overnight at 4 °C with primary antibodies: PRLR (1:1000, Thermo, 35-9200), G6PD (1:2000, Proteintech, 25413-1-AP), TKT (1:2000, Proteintech, 66016-1-Ig), p-MST1/2 (1:1000, ab79199), MST1 (1:1000, CST, #14946), p-LATS1 (1:1000, CST, #9157), LATS1/2 (1:1000, Bethyl Laboratories, Montgomery, TX), , p-YAP (1:1000, CST, #13008), YAP (1:1000, Abcam, ab52771), TEAD1 (1:1000, Abcam, ab133533), p-AKT (1:1000, CST, #4322), p-ERK1/2 (1:1000, CST, #9100), AKT (1:1000, CST, #2920), ERK1/2 (1:1000, CST, #2920), ACTB (1:1000, Abways, AB0035). Then the membrane was washed three times in TBST (10 mins each time) at room temperature, and incubated with secondary antibodies: goat-anti-mouse (1:10000, Abways, AB0102), goat-anti-rabbit (1:10000, Abways, AB0101) at room temperature for 1hr. Finally, the membrane exposure was performed using Enhanced Chemiluminiscence (ECL) by the Bio-Rad system.

PRL stimulation and PRLR inhibition assay

For PRL stimulation in cell proliferation assay, cells were cultured in media containing 2% FBS and treated with Vehicle or reconstitute PRL (final concentration: 0.5μg/ml) for 96 hrs. PRL was supplemented every 24 hrs as the same final concentration.

For PRLR inhibition in cell proliferation assay, cells were cultured in completed media and treated with vehicle or PRLR antibody (MAB1167, Novus, final concentration: 3μg/ml) for 96 hrs. PRLR antibody was supplemented every 24 hrs as the same final concentration.

For PRL stimulation in Western blotting assay, cells were cultured in FBS-free media for at least 2 hrs in advance. Then PRL (final concentration: 0.5μg/ml) was added into media lasting for 0, 5, 15, 30, 60 and 120 mins, respectively.

Cell proliferation, colony formation assay. For cell proliferation assay, cells were seeded at 2×10^3 cell/well in 96-well plates and cultured at 37 °C. Then 10% (v/v) CCK-8 (Dojindo, Kumamoto, Japan) was added to the wells and the plates were incubated at 37 °C for 1 hr. Absorbance was measured at 450 nm using a Power Wave XS microplate reader (Bio-Tek, Winooski, VT) to analyze cell viability. This experiment was performed as five biological replicates for each group and repeated twice.

For colony formation, cells were seeded at 2×10^4 cells/well in 6-well plates in culture medium and cultured at 37 °C for 2-3 weeks. Cells were stained with crystal violet until the colonies were visualized by naked eyes. The number of colonies was calculated with ImageJ. This experiment was repeated twice.

Histology and immunohistochemistry. Routinely, slides were deparaffinized and hydrated in xylene and gradient ethanol in order. For hematoxylin and eosin (H&E) staining, slides were stained in hematoxylin and eosin according to standard protocol. For immunohistochemistry, antigen retrieval was performed by boiling the slides in citrate sodium buffer for 15 mins. Next, endogenous peroxidase was blocked by incubating the slides in 0.3% (v/v) hydrogen peroxide methanol solution for 20 mins at 37 °C. Then slides were blocked by 10% BSA at room temperature for 1 hr, and incubated with primary antibodies at 4°C overnight. After HRP-conjugated secondary antibodies incubation and DAB staining, slides were counterstained with hematoxylin.

Cell transfection and lentivirus constructs. For usual cell transfection assay, cells were seeded at 60%-70% confluence in plates or dishes. The shRNA against PRLR and scrambled sequences were purchased from GenePharma (Shanghai, China), and overexpressed-PRLR plasmids were purchased from (GeneCopoeia, USA). Lentivirus particles were generated using a three-plasmid-system (pPACKH1-GAG, pPACKH1-REV and pVSV-G) preserved by our lab. Lentivirus

packaging was performed in 293T cells with co-transfection of shRNA or plasmids via Lipofectamine® 2000 Reagent according to the protocol. For lentivirus transfection assay, cells were infected with reconstructed lentivirus for 24 hrs. Then cells were treated with 1 μg/ml puromycin (Gibco, A1113802) for more than 7 days. The transfection efficiency was detected later by Western blot.

NADPH and $NADP^+$/NAPDH ratio measurement. Intracellular NADPH and $NADP^+$/NAPDH ratio were measured using cell lysates according to manufacturer's instruction provided by NADPH assay kit (Biovision, #K347). The values were normalized to the protein concentration.

Luciferase reporter assay. Luciferase reporter assays were performed using plasmids as following: PGL3B-vector, PGL3B-G6PD/TKT (containing G6PD/TKT promoter region) and PGL3B-mG6PD/TKT (containing G6PD/TKT mutant promoter region). Overexpressed-TEAD1 AsPC-1 and BxPC-3 cells were transfected with PGL3B-promoter vector and Renilla luciferase expression plasmid. After 24 hrs, the cells were collected and subjected to a Dual Luciferase Reporter Assay System (Promega).

Quantitative real-time PCR. Total RNA was extracted using RNAiso plus (Takara, Code No. 9108) and reverse transcribed with PrimeScript RT-PCR kit (TaKaRa, Code No. RR036A) according to the protocols. Real-time PCR was performed using 2x SYBR Green qPCR Master Mix (bimake, Cat#: B21202) on a 7500 Real-Time PCR system (Applied Biosystems, Foster City, CA) as following thermal cycling settings: 1 initial cycle at 95 ℃ for 10 mins, followed by 40 cycles of 15 secs at 95 ℃ and 34 secs at 60 ℃. All the primers used for Real-time PCR were purchased from TsingKe and sequences were listed in supplementary Table.

YAP-mutant rescue experiments. Mutant plasmids pcDNA3.1-YAP (S127A) and control plasmid pcDNA3.1 were kept in our laboratory. For rescue of PRL stimulation effects, AsPC-1 cells and BxPC-3 cells were treated with PRL as mentioned above for 24 hrs, then plasmids pcDNA3.1-YAP (S127A) transfection was performed as protocol with PRL treatment all the time. After culturing for 48 hrs, treated cells were used for immunofluorescence assay, LC-MS analysis and EdU incorporation assay.

EdU incorporation assay. Cells were treated previously and cultured with EdU (10 μM) for 2 hrs, then fixed with 4% paraformaldehyde for 30 mins and permeabilizated with 0.3% Triton-X100 for 15 mins. Next cells were incubated with Click Additive Solution according to

manufacturer's protocol (BeyoClick™ EdU Cell Proliferation Kit with Alexa Fluor 488) for 30 mins at room temperature. Finally, cells were counterstained with DAPI or Hoechst33342.

Supplementary figures and legends

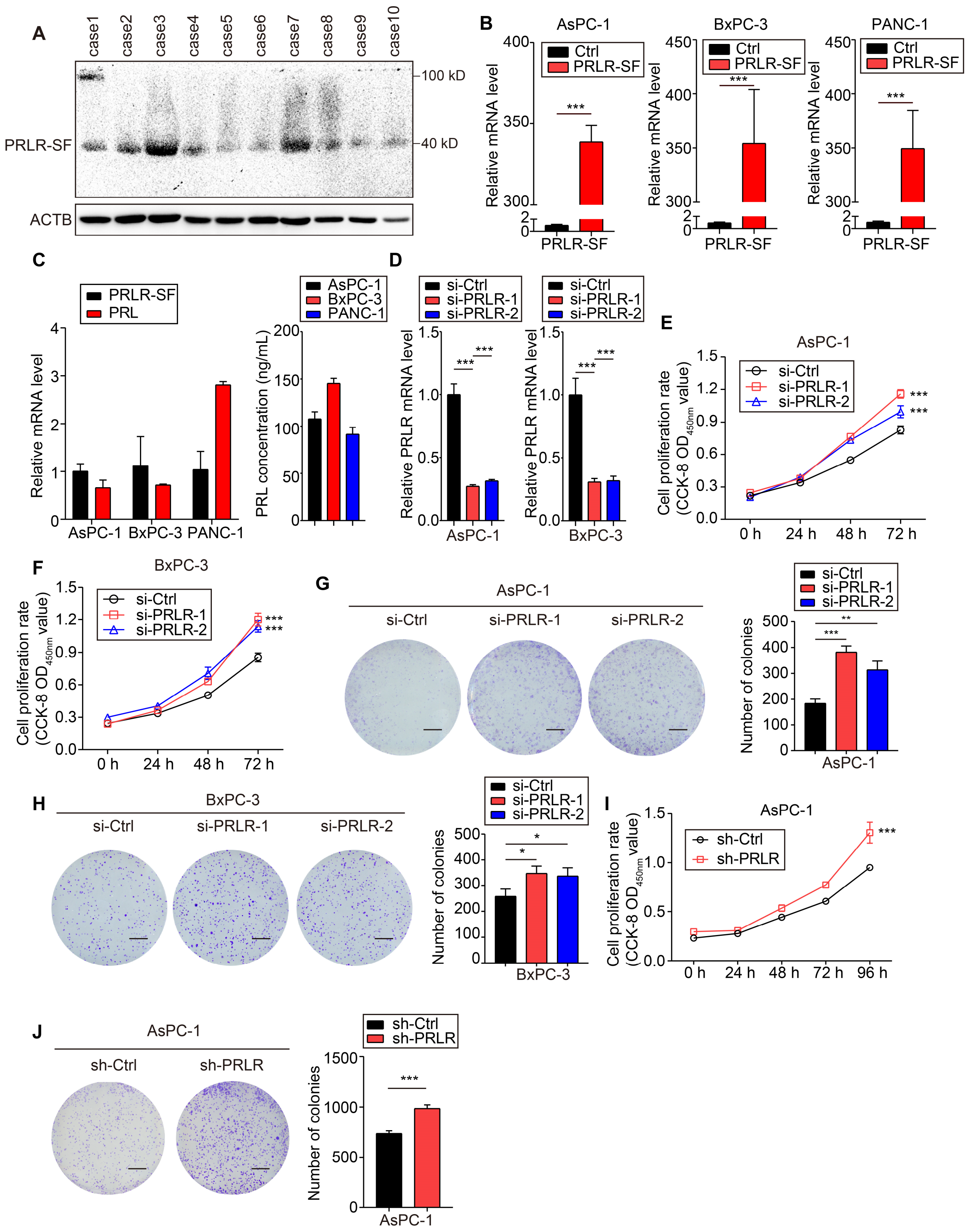

Figure S1. PRLR knockdown promotes PDAC cells growth. (**A**) Immunoblot analyses for PRLR protein expression of 10 patient-derived PDAC tissues from Ren Ji hospital. (**B**) The overexpress efficiency of PRLR-SF in AsPC-1, BxPC-3 and PANC-1 cells. n = 3, *P* values were determined by unpaired *t* test. (**C**) Real-time qPCR analyses for relative expression level of PRL

in AsPC-1, BxPC-3 and PANC-1 cells, respectively. n = 3, *P* values were determined by unpaired *t* test. (**D**) The interference efficiency of PRLR in AsPC-1 and BxPC-3 cells. n = 3, *P* values were determined by unpaired *t* test. (**E**, **F**) Proliferation assays of AsPC-1 (**E**) and BxPC-3 (**F**) cells transfected with negative control siRNA (si-Ctrl) or two *PRLR*-targeting siRNAs (si-PRLR-1 and si-PRLR-2) (n = 5). *P* values were determined by 2-way ANOVA. (**G**, **H**) Clonogenicity of AsPC-1 (**G**) and BxPC-3 (**H**) cells transfected with si-Ctrl, si-PRLR-1 and si-PRLR-2 respectively (n = 3); scale bar=500 mm. *P* values were determined by unpaired *t* test. (**I**) Proliferation of AsPC-1 cells infected with sh-Ctrl or sh-PRLR (n = 5). *P* values were determined by 2-way ANOVA. (**J**) Clonogenicity of sh-Ctrl AsPC-1 vs sh-PRLR AsPC-1 (n = 3); scale bar=500 mm. *P* values were determined by unpaired *t* test. *P < 0.05, **P < 0.01, ***P < 0.001.

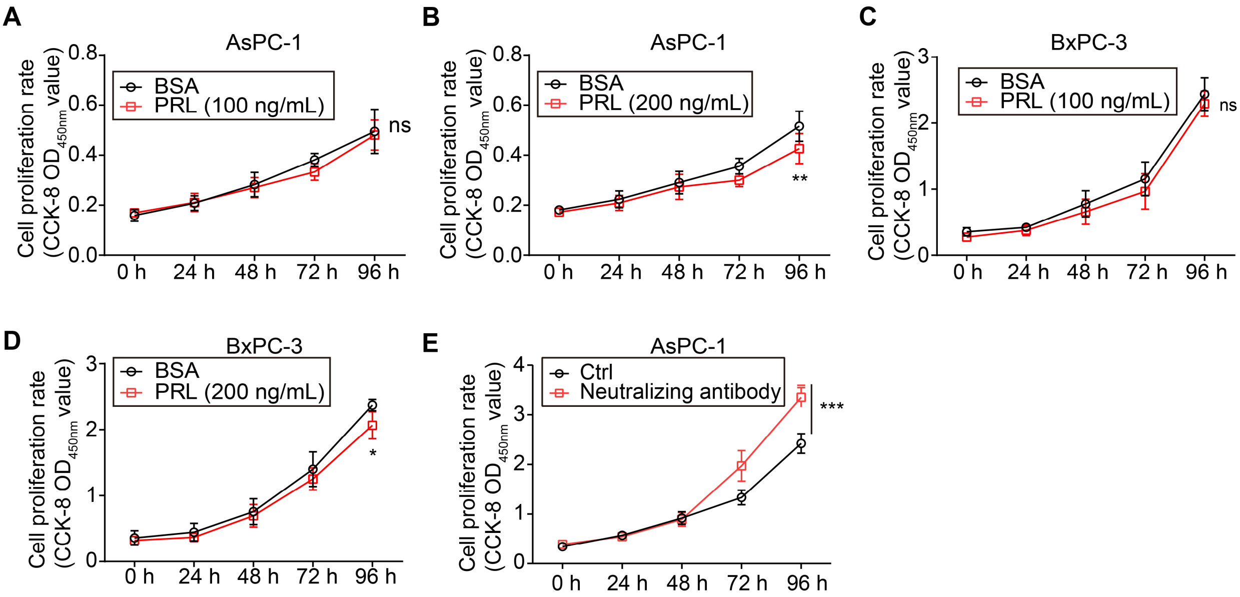

Figure S2. PRL inhibits PDAC cell growth. (**A**, **B**) Proliferation of AsPC-1 cells incubated with 100 ng/ml (**A**) or 200 ng/ml (**B**) PRL for 96 hrs (n = 5). *P* values were determined by unpaired *t* test. (**C**, **D**) Proliferation of BxPC-3 cells incubated with 100 ng/ml (**C**) or 200 ng/ml (**D**) PRL for 96 hrs (n = 5). *P* values were determined by unpaired *t* test. (**E**) Proliferation of AsPC-1 cells incubated with a control antibody (Ctrl) or a neutralizing antibody against PRLR for 96 hrs (n = 5). *P* values were determined by 2-way ANOVA. *P < 0.05, **P < 0.01, ***P < 0.001.

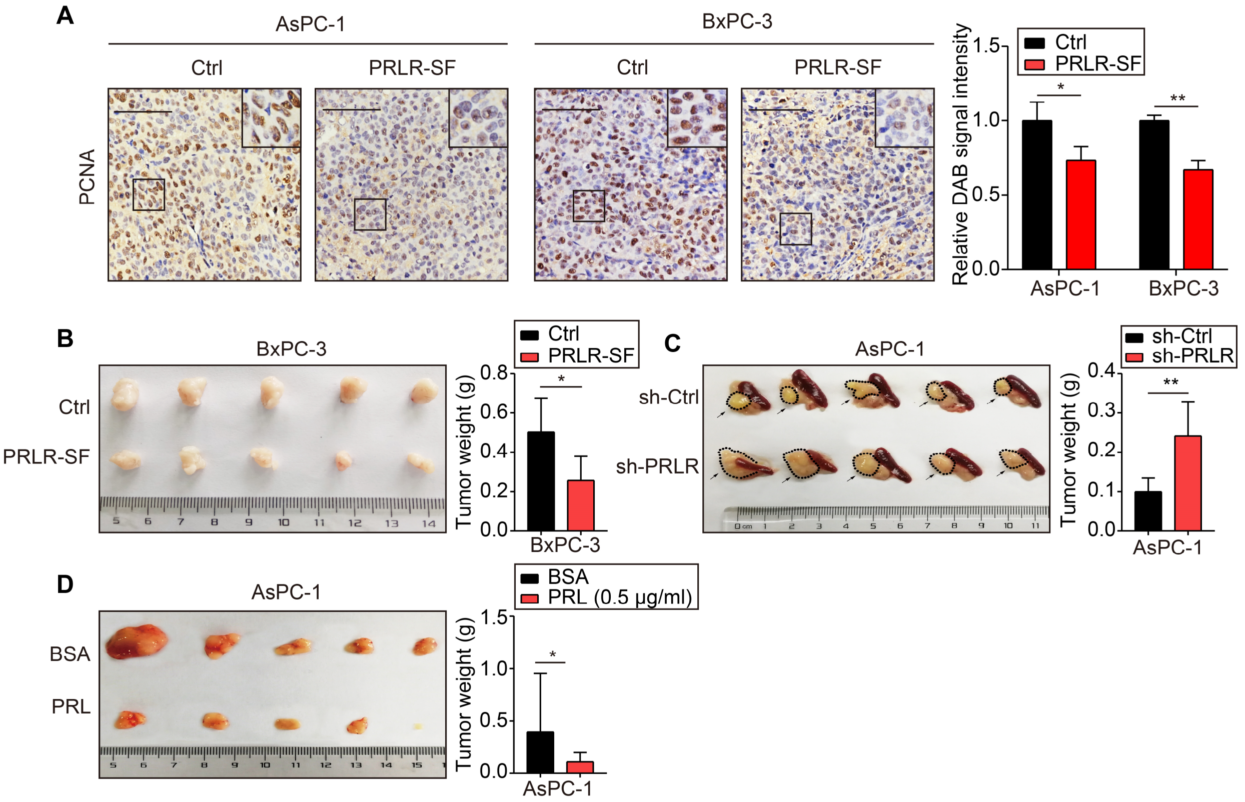

Figure S3. PRLR-SF contributes to suppression of PDAC cells growth. (**A**) Left, representative images of immunohistochemical analysis of PCNA in orthotopic xenograft tumors grown following implantation of AsPC-1 cells or subcutaneous xenograft tumors grown following implantation of BxPC-3 cells. Scale bar=100μm. Right, quantification of PCNA DAB signal intensity. n = 3, *P* values were determined by unpaired *t* test. (**B**) Tumors from subcutaneous xenografts of nude mice implanted with BxPC-3 cells infected with Lenti-Ctrl or Lenti-PRLR-SF. Tumor weight is shown (n = 5). *P* values were determined by unpaired *t* test. (**C**) Tumors with adjacent pancreas and spleen from orthotopic tumors grown in nude mice from implanted sh-Ctrl AsPC-1 or sh-PRLR AsPC-1 cells; tumor weight is shown (n = 5). *P* values were determined by unpaired *t* test. (**D**) Final xenograft tumors from nude mice, grown from subcutaneous implantations of AsPC-1 cells incubated with 0.1%BSA or PRL (0.5 μg/mL) for 10 days; final tumor weight is shown (n = 5). *P* values were determined by unpaired *t* test. * $P < 0.05$, ** $P < 0.01$.

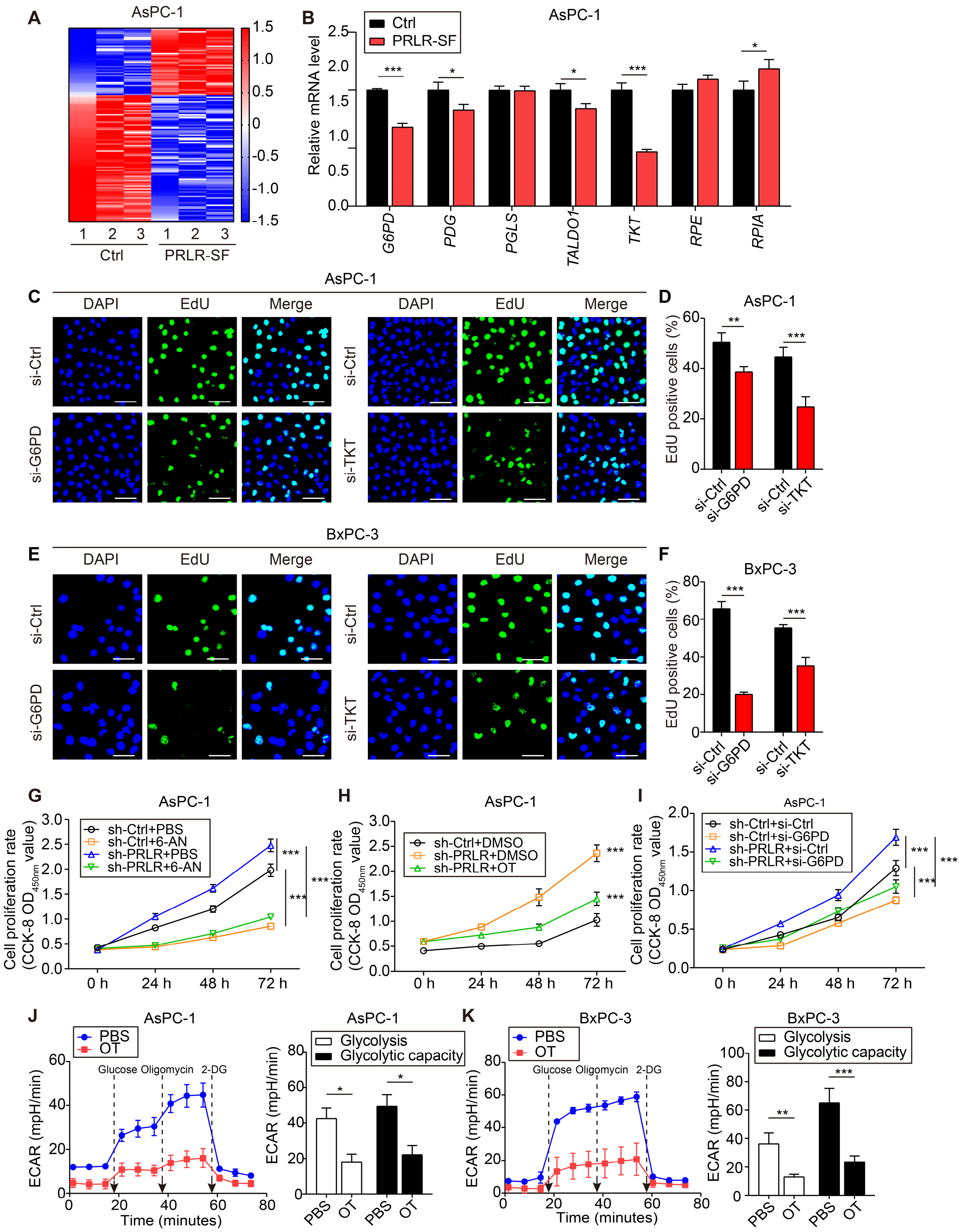

Figure S4. PRLR-SF suppresses PDAC cell proliferation through reducing G6PD and TKT expression. (**A**) Heatmap analyses for whole-genome expression profiles of AsPC-1 cells (Ctrl or overexpressing PRLR-SF). (**B**) Histograms for relative mRNA levels encoding 7 proteins in the PPP in AsPC-1 cells (Ctrl or overexpressing PRLR-SF; n = 3). *P* values were determined by

unpaired *t* test. (**C**, **D**) Representative images of DNA synthesis, detected by EdU incorporation (green) in AsPC-1 cells interfered with si-G6PD or si-TKT. DAPI staining indicates nuclei (blue). Histograms show percentages of EdU-positive cells (n = 3). (**E**, **F**) Representative images of DNA synthesis, detected by EdU incorporation (green) in BxPC-3 cells interfered with si-G6PD or si-TKT. DAPI staining indicates nuclei (blue). Histograms show percentages of EdU-positive cells (n = 3). (**G**, **H**) The effects of 6-AN (6-aminonicotinamide) (**G**) or OT (oxythiamine) (**H**) on cell proliferation of sh-Ctrl and sh-PRLR AsPC-1 cells (n = 5). *P* values were determined by 2-way ANOVA. (**I**) The effects of si-G6PD on cell proliferation of sh-Ctrl and sh-PRLR AsPC-1 cells (n = 5). *P* values were determined by 2-way ANOVA. (**J**, **K**) Effects of the TKT inhibitor OT on ECAR in AsPC-1 (**J**) and BxPC-3 (**K**)cells. Glycolysis and glycolytic capacity were calculated. *P* values were determined by unpaired *t* test. *P < 0.05, **P < 0.01, ***P < 0.001.

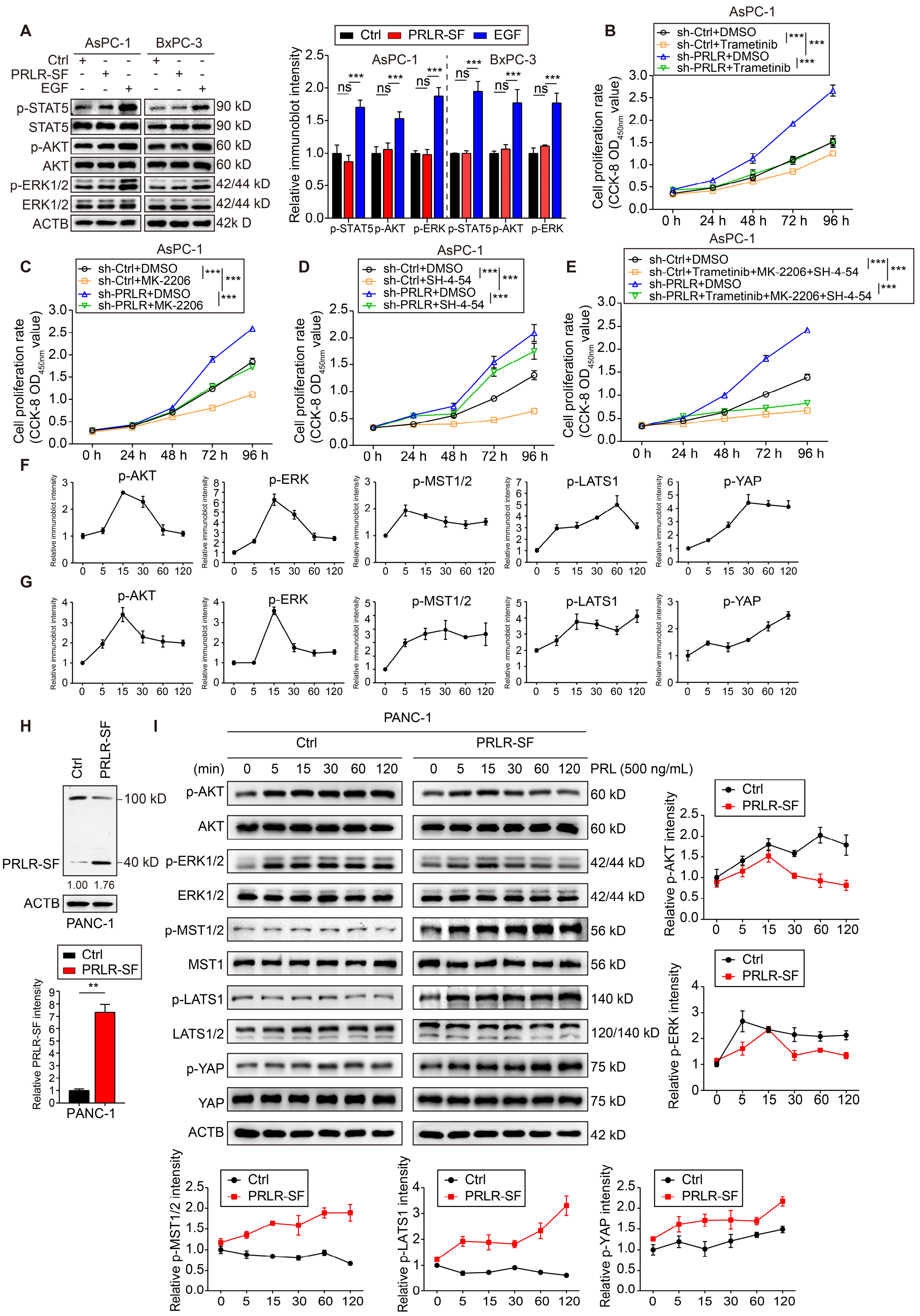

Figure S5. The effects of PRLR-SF on proliferation of PDAC cells are not affected by STAT5,

AKT and ERK1/2 but Hippo signaling pathway.

(**A**) Left, immunoblot analyses for phosphorylated (p) and total STAT5, AKT and ERK1/2 using AsPC-1 and BxPC-3 cells (Ctrl, overexpressing PRLR-SF and stimulated with 200 ng/mL EGF). Right, immunoblotting quantification of p-STAT5, p-AKT and p-ERK. n = 3 each group, *P* values were determined by unpaired *t* test. (**B-E**) The effects of Trametinib (10 μM) (**B**), MK-2206 (500 nM) (**C**), SH-5-54 (1.5 μM) (**D**) and their combinations (**E**) on cell proliferation of sh-Ctrl and sh-PRLR AsPC-1 cells (n = 5). *P* values were determined by 2-way ANOVA. (**F**, **G**) Immunoblotting quantification of p-AKT, p-ERK, p-MST1/2, p-LATS1, p-YAP in AsPC-1 cells (**F**) and BxPC-3 (**G**) cells incubated with 0.5 μg/mL prolactin (PRL) for 0-120 mins in serum-free media. n = 3. (**H**) The overexpress efficiency of PRLR-SF protein in PANC-1 cells. n = 3, *P* values were determined by unpaired *t* test. (**I**) Immunoblots analyses for levels and phosphorylation (p) of AKT, ERK1/2, and proteins in the Hippo signaling pathway in Ctrl- or PRLR-SF-overexpressed PANC-1 cells incubated with 0.5 μg/mL prolactin (PRL) for 0-120 mins in serum-free media. Counterpart immunoblotting quantifications of p-AKT, p-ERK, p-MST1/2, p-LATS1, p-YAP were shown. $^{**}P < 0.01$, $^{***}P < 0.001$.

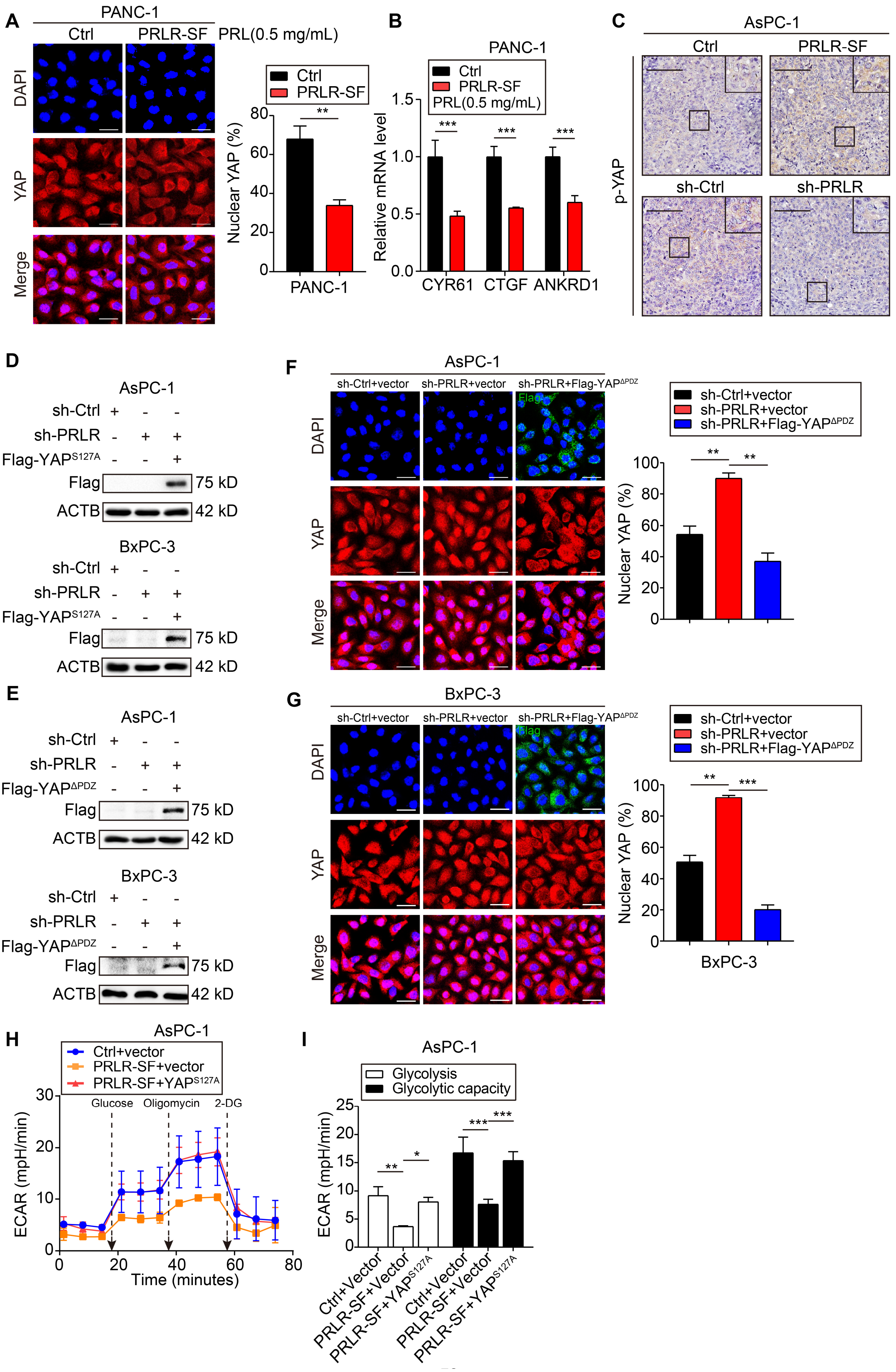
A
PANC-1
Ctrl
PRLR-SF
PRL(0.5 mg/mL)
DAPI
YAP
Merge
Ctrl
PRLR-SF
Nuclear YAP (%)
**
PANC-1
B
PANC-1
Ctrl
PRLR-SF
PRL(0.5 mg/mL)
Relative mRNA level
***
CYR61
CTGF
ANKRD1
C
AsPC-1
Ctrl
PRLR-SF
sh-Ctrl
sh-PRLR
p-YAP
D
AsPC-1
sh-Ctrl
sh-PRLR
Flag-YAPS127A
Flag
75 kD
ACTB
42 kD
BxPC-3
E
Flag-YAPΔPDZ
F
AsPC-1
sh-Ctrl+vector
sh-PRLR+vector
sh-PRLR+Flag-YAPΔPDZ
Flag
G
BxPC-3
H
AsPC-1
Ctrl+vector
PRLR-SF+vector
PRLR-SF+YAPS127A
Glucose
Oligomycin
2-DG
ECAR (mpH/min)
Time (minutes)
I
AsPC-1
Glycolysis
Glycolytic capacity
Ctrl+Vector
PRLR-SF+Vector
PRLR-SF+YAPS127A

Figure S6. Hippo signaling pathway is activated by PRLR-SF in PDAC cells. (**A**) Representative immunofluorescence images of PANC-1 cells infected with Lenti-control (Ctrl) or Lenti-PRLR-SF. Nuclei labeled in blue, YAP labeled in red, and combined images below. Scale bar = 25 μm. Quantitative histograms show percentages of nuclear YAP in each cell type (n = 3). (**B**) Relative levels of *CYR61*, *CTGF*, and *ANKRD1* mRNAs in control (Ctrl) PANC-1 cells vs those that overexpress PRLR-SF (n = 3). (**C**) Representative immunohistochemical staining images for detecting p-YAP level using orthotopically implanted tumors formed by Ctrl and overexpressing PRLR-SF AsPC-1 cells (top) and sh-Ctrl and sh-PRLR AsPC-1 cells (bottom). (**D**, **E**) Immunoblot analyses for the effects of overexpressing Flag-YAP^{S127A} (**D**) or $YAP^{\Delta PDZ}$ (**E**) using AsPC-1 cells (top) and BxPC-3 cells (bottom). (**F**, **G**) Representative immunofluorescence images of AsPC-1 cells (**F**) or BxPC-3 cells (**G**) infected with Lenti-sh-control (Ctrl) or Lenti-sh-PRLR, and transfected transiently with empty vectors (vector) or vectors that overexpress Flag-$YAP^{\Delta PDZ}$. Flag labeled in green, nuclei labeled in blue, YAP labeled in red, and combined images below. Scale bar = 25 μm. Quantitative histograms show percentages of nuclear YAP in each cell type (n = 3). H, **I**) Effects of transfecting YAP^{S127A} on ECAR in AsPC-1 cells (Ctrl and overexpressing PRLR-SF). Glycolysis and glycolytic capacity are calculated. *P* values were determined by unpaired *t* test. *P < 0.05, **P < 0.01, ***P < 0.001.

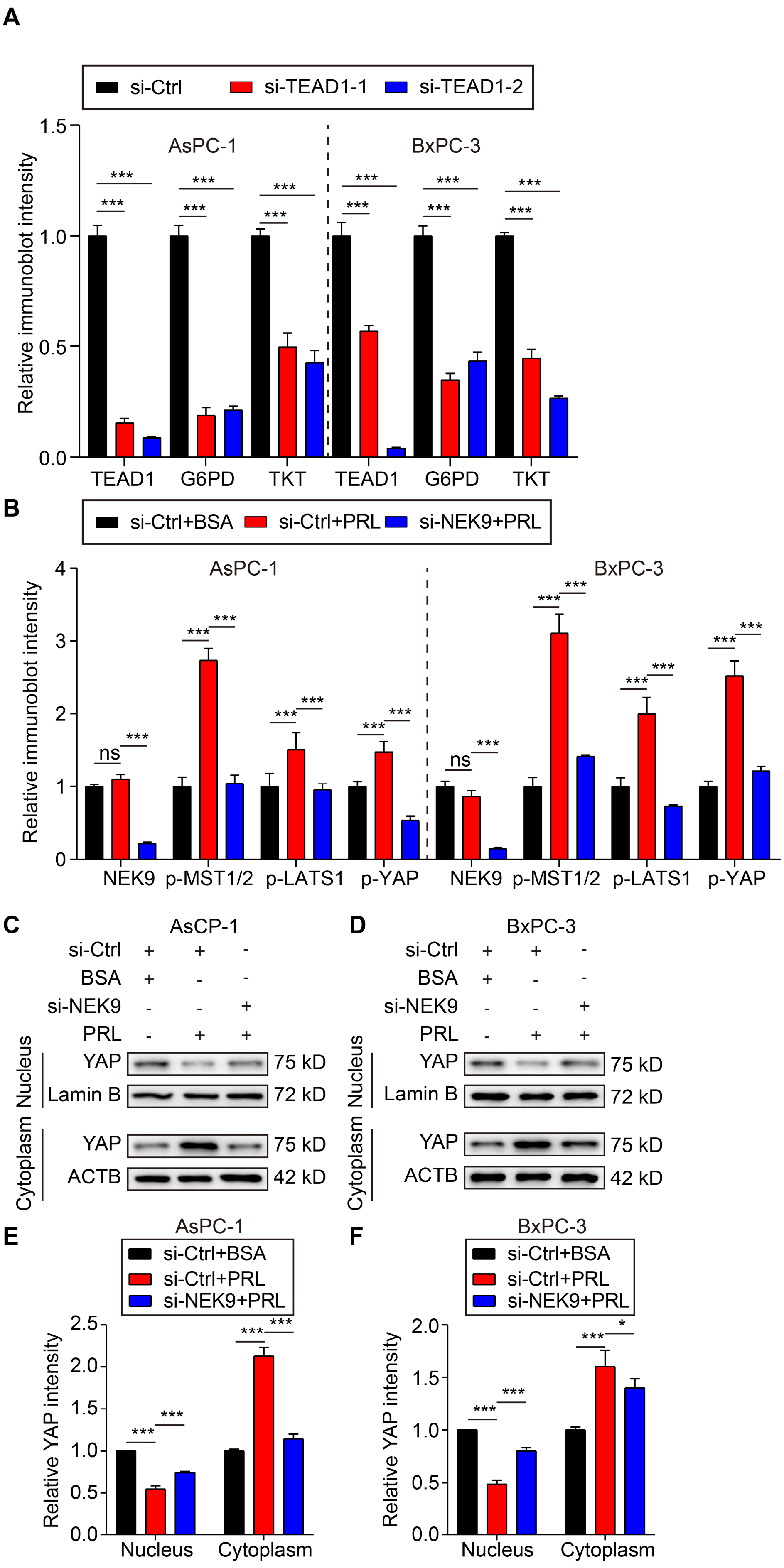

A
si-Ctrl
si-TEAD1-1
si-TEAD1-2
Relative immunoblot intensity
1.5
1.0
0.5
0.0
AsPC-1
BxPC-3
***
TEAD1
G6PD
TKT
TEAD1
G6PD
TKT
B
si-Ctrl+BSA
si-Ctrl+PRL
si-NEK9+PRL
Relative immunoblot intensity
4
3
2
1
0
AsPC-1
BxPC-3
ns
***
NEK9
p-MST1/2
p-LATS1
p-YAP
NEK9
p-MST1/2
p-LATS1
p-YAP
C
AsCP-1
si-Ctrl + + -
BSA + - -
si-NEK9 - - +
PRL - + +
Nucleus
YAP 75 kD
Lamin B 72 kD
Cytoplasm
YAP 75 kD
ACTB 42 kD
D
BxPC-3
si-Ctrl + + -
BSA + - -
si-NEK9 - - +
PRL - + +
Nucleus
YAP 75 kD
Lamin B 72 kD
Cytoplasm
YAP 75 kD
ACTB 42 kD
E
AsPC-1
si-Ctrl+BSA
si-Ctrl+PRL
si-NEK9+PRL
Relative YAP intensity
2.5
2.0
1.5
1.0
0.5
0.0
***
Nucleus
Cytoplasm
F
BxPC-3
si-Ctrl+BSA
si-Ctrl+PRL
si-NEK9+PRL
Relative YAP intensity
2.0
1.5
1.0
0.5
0.0
***
*
Nucleus
Cytoplasm

Figure S7. Immunoblotting quantification for interfering TEAD1 and NEK9. (**A**) Immunoblotting quantification of TEAD1, G6PD and TKT in AsPC-1 and BxPC-3 cells interfered by si-RNAs against TEAD1. n = 3 each group, *P* values were determined by unpaired *t* test. **(B**) Immunoblotting quantification of NEK9, p-MST1/2, p-LATS1, p-YAP in AsPC-1 and BxPC-3 cells treated with si-NEK9 and PRL (0.5 μg/mL). n = 3 each group, *P* values were determined by unpaired *t* test. (**C**, **D**) Immunoblot analyses for the effects of PRL stimulation and interfering NEK9 on YAP distribution in nucleus and cytoplasm using AsPC-1 cells (**C**) and BxPC-3 cells (**D**). (**E**, **F**) Immunoblotting quantification of YAP expression level in nucleus and cytoplasm using AsPC-1 (**E**) and BxPC-3 (**F**) cells treated with si-NEK9 and PRL (0.5 μg/mL). n = 3 each group, *P* values were determined by unpaired *t* test. ***P < 0.001.

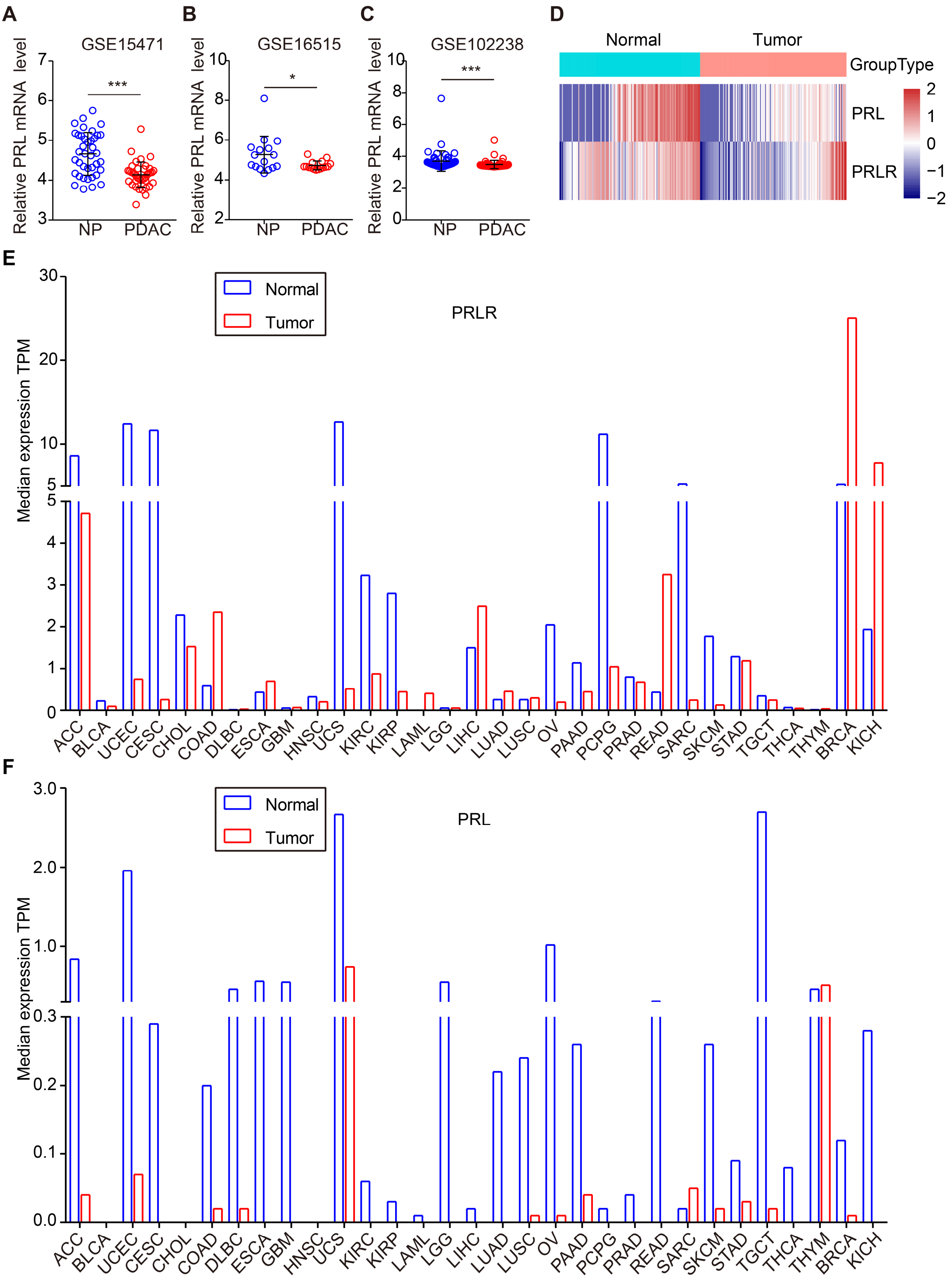

Figure S8. PRLR and PRL is downregulated in PDAC. (**A-C**) Relative *PRL* mRNA level of paired NP and PDAC tissues in GSE15471 (**A**), GSE16515 (**B**) and GSE102238 (**C**). *P* values

were determined by paired two-tailed t-test. (**D**) Heatmaps for *PRLR* and *PRL* mRNA level based on PAAD tissues of TCGA data and GTEx data. (**E**, **F**) *PRLR* and *PRL* expression profile across pan-cancer tumor tissues and paired normal tissue integrated with GTEx data (TPM: Transcripts Per Kilobase Million). *P < 0.05, ***P < 0.001.

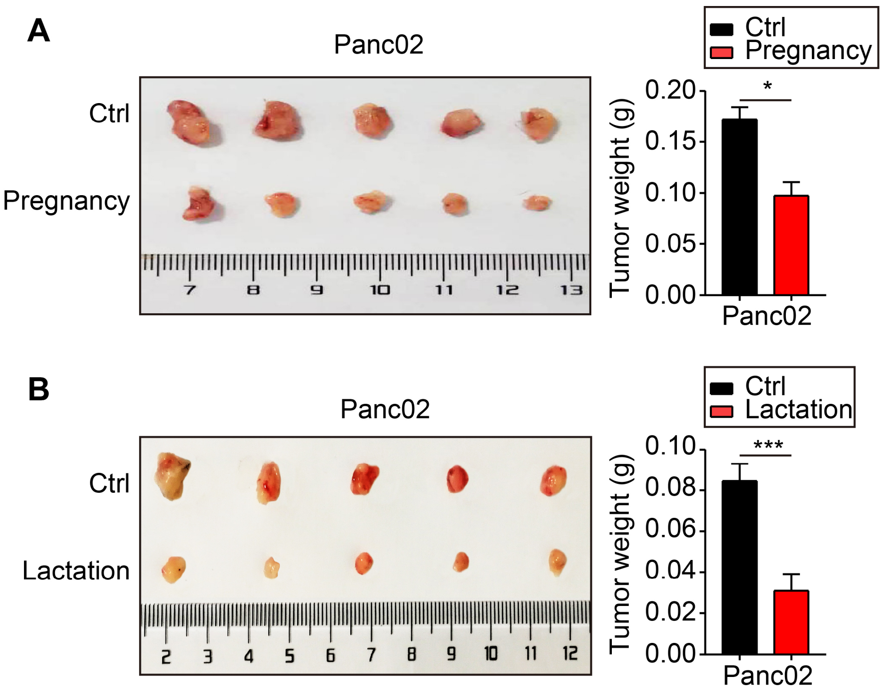

Figure S9. Pregnancy and lactation reduce PDAC cell growth. Tumors grown from subcutaneous injection of Panc02 cells in mice that were not pregnant (Ctrl) or were pregnant (**A**) or that were not lactating (Ctrl) or were lactating (**B**). Tumor weight is shown (n = 5). *P* values were determined by unpaired *t* test.

Supplementary Table 1 Primers Used for Quantitative PCR in This Article

Gene symbol	Forward sequence (5'-3')	Reverse sequence (5'-3')
H-PRLR-LF	GCTCCGCTAAACCCTTGGAT	GAGCCTGCACTTGCTTGATG
H-PRLR-SF1a	GATGCCAAGACTTTCCTCCCA	GCCTTTGTGAACACCGCAAG
H-PRLR-SF1b	GGAGTCCAGCGACCTTCATT	AGGGGTCACCTCCAACAGAT
M-Prlr-LF	ATAAAAGGATTTGATACTCAT CTGCTAGAG	TGTCATCCACTTCCAAGAACTCC
M-Prlr-SF-1	TGCATCTTTCCACCAGTTCCGGGG C	TTGTATTTGCTTGGAGAGCCAGT
M-Prlr-SF-2	ATCTTGTCCAGACTCGCTGC	TCTTCACCAGGGAAGTCAACTG
G6PD	CGAGGCCGTCACCAAGAAC	GTAGTGGTCGATGCGGTAGA
TKT	TCCACACCATGCGCTACAAG	CAAGTCGGAGCTGATCTTCCT
PGD	GACATCATCATTGACGGAGGAAA	GGGCCACGCTTCTTTGTTC
PGLS	GGAGCCTCGTCTCGATGCTA	GAGAGAAGATGCGTCCGGT
TALDO1	CAGCACAGATGCCCGCTTA	CGGCCCGGAATCTTCTTTAGTA
RPE	CAGGACCCTTTCTTTGACATGC	CAAGGCCAACCTTCATCCCAT
RPIA	GAACCTCGTCTGTATTCCCACT	CTGGGTGTCGATCCAGATCAC
CYR61	AGCCTCGCATCCTATACAACC	TTCTTTCACAAGGCGGCACTC
CTGF	AAAAGTGCATCCGTACTCCCA	CCGTCGGTACATACTCCACAG
ANKRD1	CACTTCTAGCCCACCCTGTGA	CCACAGGTTCCGTAATGATTT
RPS18	TGCGAGTACTCAACACCAACA	GCATATCTTCGGCCCACA

Supplementary Table 2 Primers Used for CHIP-PCR of the G6PD and TKT Promoter

Gene symbol	Sequence (5'-3')	Location
G6PD	GGCGGACTCAGACTTCTCTCCGGAG	-156/-122
	ACGTGAGAAGGGTGGGAGCGGCGGA	+55/+76
TKT	CCGCAGTGGTGGCCTGGATGGAGCT	+14/+38
	CCTCCTCCCCCTGCCCCGCCTCTCC	+245/+269

Supplementary Table 3 The sequence of siRNA/shRNA

siRNA/shRNA Genes	Sense sequence (5'-3')
si-PRLR-1	GUGGCUUUGAAGGGCUAUATT
si-PRLR-2	GGGCAAGUCUGAAGAACUATT
Sh-PRLR	CCGGAGCAGCTGATTCCAGAAC ATGTTCTGGAATCAGCTGCTTTTTG
si-G6PD-1	GAGAGUGGGUUUCCAGUAUTT
si-G6PD-2	GCGUUAUCCUCACCUUCAATT
si-TEAD1-1	CUGCCAUUCAUAACAAGCUTT
si-TEAD1-2	GGCAUGCCAACCAUUCUUATT
si-NEK9-1	CCAUAAAGCUGGAAUCCUUTT
si-NEK9-2	CCUCCUAUCGACAGCCAAATT
